# Non-parametric differential methylation analysis characterizes histotype-specific promoter regions in epithelial ovarian cancer

**DOI:** 10.1080/15592294.2026.2720990

**Published:** 2026-09-02

**Authors:** Hugo Swenson, Ella Ittner, Lucas Werner, Elisabeth Werner Rönnerman, Szilárd Nemes, Toshima Z. Parris, Khalil Helou

**Affiliations:** a Department of Oncology, Institute of Clinical Sciences, Sahlgrenska Academy, University of Gothenburg, Gothenburg, Sweden; b Sahlgrenska Center for Cancer Research, Sahlgrenska Academy, University of Gothenburg, Gothenburg, Sweden; c Department of Clinical Pathology, Region Västra Götaland, Sahlgrenska University Hospital, Gothenburg, Sweden

**Keywords:** Epigenetics, biomarker discovery, method optimization, predictive classification

## Abstract

Epithelial ovarian cancer (EOC) is a heterogenous disease with frequent late-stage diagnosis and high mortality rates, for which no reliable screening tests exist. In recent years, epigenetic biomarkers in the form of DNA methylation in CpG-rich regions have gained increased attention in the scientific community due to their robust nature and accessibility, allowing for diagnosis without the need for invasive surgery. In this study, we investigated the aberrant methylation of promoter regions in early stage EOC through non-parametric methods, with the purpose of characterizing candidate epigenetic biomarkers. The approach was used on a cohort of early stage EOC samples, and results were compared to existing programs for differential methylation. Significant regions were then used to construct a CpG panel for stratifying EOC histotypes through predictive classification in external data. Identified promoter regions were highly reproducible across cohorts, and the constructed CpG model stratified histotypes in external cohorts through predictive classification. Comparisons against other DMP and DMR callers showed a degree of homogeneity between results but also revealed promoter regions that were overlooked despite clear signs of aberrant methylation. Finally, EOC histotypes were found to differ in their methylation distribution types, and results indicate that methods sensitive to non-normally distributed data may be poorly suited to compare groups with different distribution types. The non-parametric approach identified aberrantly methylated promoter regions that were highly reproducible across cohorts. Results from predictive classification indicate that these regions may be useful for the purpose of EOC histotype stratification.

## Introduction

Ovarian cancer (OC) is a heterogenous disease, which is responsible for approximately 20% of cancer-related deaths in women while only accounting for about 2% of female cancers [1,2]. Malignant epithelial OCs (EOC), which make up 90% of the total OC cases, are stratified into five primary histological subtypes; high-grade serous carcinoma (HGSC, 70% of EOC cases), low-grade serous carcinoma (LGSC, 5% of EOC cases), clear cell carcinoma (CCC, 10% of EOC cases), endometrioid carcinoma (EC, 10% of EOC cases), and mucinous carcinoma (MC, 3% of EOC cases [3]. Despite distinct differences in morphology, incidence rates, patient clinical outcome and molecular profiles, treatment of EOC is typically administered through a standardized protocol of debulking surgery combined with platinum-based chemotherapy as if it were a homogenous entity [4,5]. When detected and treated during the early stages of carcinogenesis, EOC has a significantly more favorable prognosis (stages I-II; 90–70% survival rate) than when it is detected during the later stages (stages III, IV; 40–30% survival rate). However, the disease is often asymptomatic in the early stages, and current screening methods for EOC are lacking [6]. More effective methods and biomarkers reflecting the biological characteristics of the primary tumor are therefore needed to improve early stage diagnosis and histotype stratification of EOC to help guide treatment decision and improve patient outcomes [7,8].

Broad epigenetic alterations, including global hypomethylation of the genome and focal hypermethylation of the promoter region of tumor suppressor genes, are known hallmarks of cancer that increase in frequency during cancer progression [9]. The addition of methyl groups to CpG sites (hypermethylation) located in CpG-islands in the promoter region of a gene is known to directly regulate gene expression by preventing transcription factors from binding to the promoter region and stabilizing the nucleosome. Inversely, the loss of methyl groups from hypermethylated CpG sites in a promoter region (hypomethylation) is typically associated with an increase in transcription of the associated gene [10]. As DNA-methylation is furthermore a chemically stable process present in the cell-free DNA of bodily fluids, DNA methylation-based biomarkers hold great potential for accessible tumor-based fingerprinting without the need for invasive surgery and have caught the attention of the scientific community in recent years. Given that EOC histotypes are distinct entities both epigenetically and transcriptionally [11], aberrant methylation patterns in functionally important genomic loci have gained interest in recent years as a promising source for candidate biomarkers for the purpose of disease monitoring and detection [12].

## Analysis of DNA methylation

To study DNA methylation, the probe hybridization-based Illumina Infinium (450k and EPIC) microarrays are a popular choice, providing a reasonable tradeoff between resolution and cost [13,14]. The platforms are based on probes that correspond to the genomic position of a CpG site, where after bisulphite conversion, the methylated state of each given probe is measured using fluorescence through labeled nucleotides [15]. The resulting methylated and unmethylated signal intensities for the CpG are then converted into β-values or *M*-values to quantify the level of methylation:

$$\beta =\frac{M_{i}}{M_{i}+U_{i}+O)},M_{i}=\mathrm{Methylatedintensity},U_{i}=\mathrm{Unmethylatedintensity},O=\mathrm{Offset}\left(\mathrm{standard}=100\right)$$

$$M=\log_{2}\left(\frac{\beta }{1-\beta }\right)$$

For statistical purposes *M*-values are preferable to those of β-values as the latter experience heteroscedasticity in the upper and lower ranges, which can negatively impact statistical models, whereas *M*-values are approximately homoscedastic. However, *M*-values are comparatively more difficult to interpret biologically, and consequently β-values, which range between 0 and 1 (0 representing a completely hypomethylated state, 1 representing a fully hypermethylated state) are often used to measure effect sizes [16].

To date, several methods have been developed for the detection of differentially methylated CpG sites/probes (DMCs/DMPs) and differentially methylated genomic regions (DMRs), using either *M*-values, β-values or a combination of both. In DMP analysis, individual CpG sites are evaluated between groups of interest using statistical tests for significance and effect size with the aim of identifying differentially methylated probes. In contrast, DMR detection methods aim to identify genomic regions showing aberrant methylation patterns (i.e., containing multiple DMPs), with what constitutes such a region differing based on the method used. As the biological context of DNA methylation varies depending on the genomic location, pooling adjacent CpG sites into genomic regions provides clearer biological interpretability when compared to that of individual sites and has been shown to yield greater discriminatory power [17]. However, CpG sites are not evenly spaced on the Infinium arrays and are known to exhibit co-methylation depending on nucleotide proximity. Genomic regions of interest with a defined length and location could consequently see their significance influenced when attempting to uncover differential methylation due to sparse CpG site coverage, spatial covariation or high within group variance [18–20].

## Parametric and non-parametric methods

Parametric methods refer to statistical methods that make assumptions regarding the shape of the distribution of the data on which they are applied. Namely, that the data is continuous, homoscedastic and approximately normally distributed. Offering high statistical power when their assumptions are met, but with the risk of providing inaccurate measures for significance or effect-size when violated [21]. Non-parametric methods make no such assumptions of the data, instead relying on signs and ranks of observations rather than exact measurements. Although the assumption of normality is valid in many cases, it has been shown to be frequently violated in data from biological origins, which often display heterogeneity due to both biological and technical sources of variation (Midway and White [22]. Given that β-values are representative of methylation levels corresponding to a methylated and unmethylated state in a bounded range between 0 and 1, the distribution of values often takes on a bimodal or skewed shape [16]. Although *M*-values are more appropriate for statistical inference due to their unbounded scale and comparatively superior homoscedacity, in real-world data they often fail to meet the assumptions of a Gaussian model [23]. Consequently, parametric methods, which assume that methylation data is normally distributed such as the arithmetic mean or students t-test, may be at risk of having their assumptions frequently violated.

The non-parametric approach shown in this study attempts to circumvent the statistical pitfalls of non-normally distributed data through utilizing the Kruskal–Wallis followed by Dunn’s tests for multiple comparisons when testing sites for statistical significance (*M*-values). And through using Tukey’s trimean: $\mathrm{TM}=\frac{Q_{1}+2Q_{2}+Q_{3}}{4}=\frac{1}{2}\left(Q_{2}+\frac{Q_{1}+Q_{3}}{2}\right)$ when testing for biological significance (β-values). The latter calculates the central tendency of a distribution by splitting it into three quartiles with the central quartile being prioritized. Resulting in a robust estimation of the central tendency of a distribution equivalent to the mean of the sum of the median and midhinge. Unlike methods like the median or trimmed mean, TM does therefore not forsake the more extreme values in the distribution, making it suitable for smaller datasets not following the assumptions of normality [24]. Finally, to further account for dependencies of adjacent CpG sites within the same promoter region, *p*-values for histotype comparisons from Dunn’s test were aggregated through the Empirical Browns method when measuring overall significance, as they cannot be considered to be independent from one another due to their proximity within a functionally relevant region and correlated methylated states [25].

## Aims

While previous studies have shown that promoter methylation in EOC is correlated with patient outcome and can be of diagnostic use [26,27], to our knowledge no studies have investigated promoter methylation for the purpose of EOC histotype stratification. With these considerations in mind, we established a non-parametric strategy in pre-defined promoter regions. Utilizing the Brunner–Munzel test in comparisons between two groups, and the Kruskal–Wallis test followed by Dunn’s test in multi-group comparisons to measure significance. With effect size (β) measured through Tukey’s Trimean (TM). The aims of this study were to (1) identify promoter regions in EOC uniquely methylated for one histotype and (2) evaluate the impact of distribution types on significance and effect size in DNA methylation analysis of EOC. To facilitate this study, DNA methylation profiles for 86 early stage EOCs were studied, followed by validation using external EOC cohorts.

## Methods

### Patient cohorts and data acquisition

**Figure 1.**
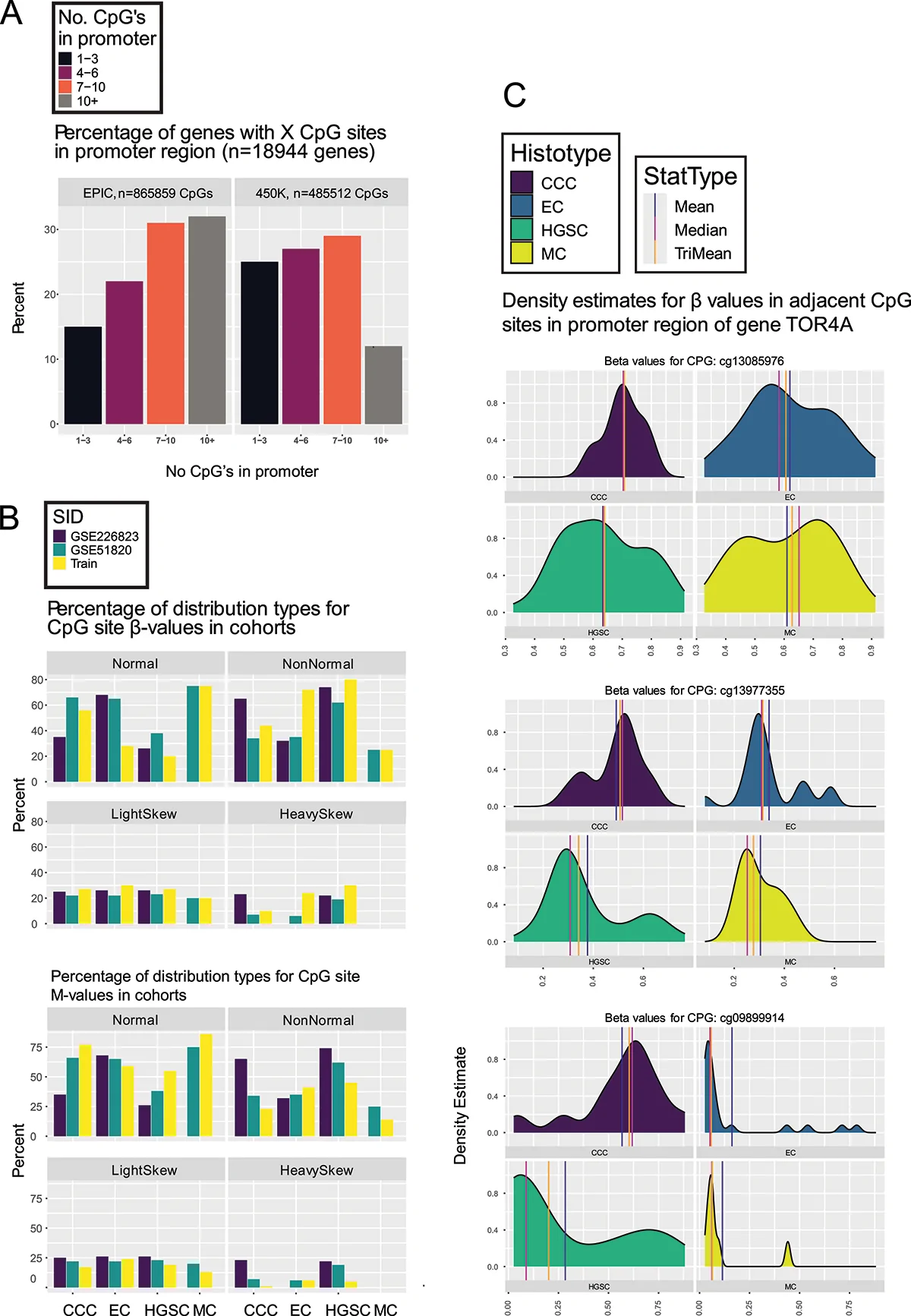
Promoter statistics for the Training cohort (A) CpG coverage of promoter regions (2000 bp upstream, 200 bp downstream of TSS) in the EPIC and 450K arrays, (B) Training and testcohort histotype β and *M*-value distribution types for all CpG sites, (C) Scaled density plots of β values for adjacent CpG-sites in the promoter region for the gene TOR4A . Np-promoter: Promoter region for a gene deemed uniquely significantly differentially methylated by the non-parametric approach described in the manuscript, CCC: Clear Cell Carcinoma, EC: Endometrioid Carcinoma, HGSC: High-Grade Serous Carcinoma, MC: Mucinous Carcinoma, TriMean: Tukeys Trimean, TSS: Transcription start site, StatType: Statistical method used to calculate the central tendency of the distribution.

To investigate epigenetic differences in the promoter regions of genes between the histotypes of early stage (stage I–II) EOC, 86 cases were chosen as the training cohort from a previous study (GSE326000/Training cohort [28], Figure 1(A)) comprised of 4/5 main EOC histotypes, i.e., HGSC (*n* = 45), MC (*n* = 7), EC (*n* = 21), and CCC (*n* = 13; Table 1). For validation, raw.idat files for seven external array-based (Infinium 450K/EPIC) EOC methylation datasets were retrieved from the Gene Expression Omnibus (GEO) repository along with their phenotypic annotations (Validation cohort: GSE51820, GSE226823, GSE133556, GSE211686, GSE263434, GSE267068, GSE155760; Table 2). From the validation cohort, two datasets with EOC samples of varying grade for the investigated histotypes, were chosen as the test cohort for predictive modeling (Test cohort: GSE51820 (4/4 histotypes), GSE226823 (3/4 histotypes); SI: Figure. S1-B, C).

**Table 1.** Clinicopathological features of the training cohort, stratified by histotype (*n* = 86).

Characteristic	All (*n* = 86)	HGSC (*n* = 45)	CCC (*n* = 13)	MC (*n* = 7)	EC (*n* = 21)
**Age at diagnosis (years)**					
Mean	63.8	65	62.3	61.7	62.6
Range	25–86	38–86	42–79	39–80	25–83
**Stage**					
I	56 (65.1)	25 (55.6)	11 (84.6)	6 (85.7)	14 (66.7)
II	30 (34.9)	20 (44.4)	2 (15.4)	1 (14.3)	7 (33.3)
**Relapse**					
Yes	31 (36.0)	17 (37.8)	4 (30.8)	4 (57.1)	6 (28.6)
Not Available	55 (64.0)	28 (62.2)	9 (69.2)	3 (42.9)	15 (71.4)
**Adjuvant chemotherapy**					
Platinum single	52 (60.5)	26 (57.8)	7 (53.9)	6 (85.7)	14 (66.7)
Platinum Combination	25 (29)	12 (26.7)	6 (46.2)	0	7 (33.3)
Non-platinum	8 (9.3)	7 (15.6)	0	1 (14.3)	0

n (% of total rounded up to one decimal point). *CCC*, clear cell carcinoma; *EC*, endometrioid carcinoma; *HGSC*, high-grade serous carcinoma; *MC*, mucinous carcinoma; *OC*, ovarian carcinoma.

**Table 2.** Histotype and microarray platform characteristics for the training and test cohorts.

Cohort name	GEO Accession ID	CCC	EC	HGSC	MC	Healthy subjects	Platform
Training	GSE326000	13	21	45	7	0	Illumina Infinium EPIC
Validation	GSE51820	13	11	53	8	0	Illumina Infinium 450K
	GSE226823	37	8	46	0	0	Illumina Infinium 450K
	GSE133556	0	0	99	0	12	Illumina Infinium EPIC
	GSE211686_1	0	0	73	0	0	Illumina Infinium 450K
	GSE211686_2	0	0	58	0	0	Illumina Infinium EPIC
	GSE263434	0	12	258	0	0	Illumina Infinium EPIC
	GSE267068	0	0	92	0	0	Illumina Infinium EPIC
	GSE155760	0	0	23	0	11	Illumina Infinium EPIC
	GSE185008_1	40	0	0	0	0	Illumina Infinium 450K
	GSE185008_2	230	0	0	0	0	Illumina Infinium EPIC

*CCC*, clear cell carcinoma; *EC*, endometrioid carcinoma; *GEO*, Gene Expression Omnibus; *Healthy*, Paired control samples in the dataset; *HGSC*, high-grade serous carcinoma; *MC*, mucinous carcinoma.

### DNA methylation preprocessing

DNA methylation microarray data for the training and validation cohorts were processed with the same bioinformatic pipeline in R (v.4.3.0 [29] using the minfi package (v.1.48.0 [30]. Samples with detection *p* > 0.01 in *n* > 5% of samples were removed from analysis alongside probes (1) with a detection *p*-value > 0.01, (2) a conversion rate < 80%, (3) overlapping single-nucleotide polymorphism (SNP) sites, (4) localized on the Y chromosome, and (5) identified as cross-reactive probes through the maxprobes package (v.0.0.2; M [31]). The remaining probes were then additionally filtered with the ChAMP package champ.filter function (v.2.32.0) [32]. β-values for the remaining probes were normalized by Noob (minfi), followed by BMIQ ChAMP).

### Analysis of methylation profiles and distribution types

CpG site hg38 probe coordinates from the Infinium MethylationEPIC v1.0 B5 manifest file for the EPIC array were mapped to genomic coordinates retrieved using the biomaRt package (v.2.58 [33], Ensembl GRCh38.p14). Additionally, coordinates from the EPIC array annotation were cross referenced with the data in the HumanMethylation450 v1.2 manifest file for the Infinium HumanMethylation450 array to create a separate hg19 annotation using biomaRt (Ensembl GRCh37.p13) genomic coordinates. Promoter regions for protein coding genes excluding the Y-chromosome were defined as ranging from 2000 bp upstream, 200 bp downstream of transcription start site (TSS). CpG site β values for the samples were classified as hypomethylated when β < 0.3, hemimethylated when 0.3 < β < 0.7 and hypermethylated when β > 0.7.

For each histotype, the frequency of CpG sites for all samples adhering to a methylation type category was calculated, and CpG site β value distribution type analysis was performed on the training and test cohort datasets using the Shapiro–Wilks test for normality through the shapiro.test function in the R stats package. Distributions showing *p* > 0.05 were deemed to be normally distributed, while distributions showing *p* < 0.05 were deemed as non-normally distributed. Skewness for a CpG site was calculated using the e1071 R package skewness function (v.1.7–16 [34], assigning CpG sites with skewness < 1 and shapiro-test *p* < 0.05 as half normally distributed, CpG sites with 1 ≤ skewness ≤2 as lightly skewed, and CpG sites with skewness > 2 as heavily skewed distributions. The process was then repeated for *M*-values. Non-parametric differential pairwise comparisons were carried out using the Brunner–Munzel test through the Brunner–Munzel R-package (v 2.0 [35]) and compared against the parametric students t-test. Similarly, to evaluate effect-size differences in pairwise comparisons, the arithmetic mean and TM were compared to one another. For comparisons against multiple groups, the Kruskal–Wallis (KW) test was used followed by Dunn’s test through the DescTools R package (v.0.99.58 [36]) using the histotype investigated as reference and was compared against the results from the same comparisons in Tukey’s test following one-way Analysis of Variance (ANOVA).

### Differential methylation analysis and external validation

To detect differentially methylated promoter regions, given that CpG sites are known to exhibit co-methylation based on nucleotide distance, for each histotype group comparison made, the unadjusted *p*-values from Dunns test were aggregated using the Empirical Browns method (EB) through the EmpiricalBrownsMethod package (v1.30.0 [37]) giving a representative measurement of significance for the entire promoter region for a reference group against the other histotypes. Additionally, unadjusted *p*-values for all CpG-site between-group comparisons made using Dunns test within the promoter region were corrected using Benjamini–Hochberg (BH), rather than correcting for the comparisons made for individual CpG sites. To determine effect sizes, CpG site β-value differences were calculated using TM in pairwise comparisons. Promoter regions were considered to be significantly differentially methylated if they contained *n* ≥ 3 overlapping CpG sites, out of which *n* ≥ 25% (minimum of 2) CpG sites had (1) KW *p* < 0.05 (BH) and additionally (2) Dunn’s adjusted *p* < 0.05 (BH) (3) an aggregated EB *p*-value < 0.05 and (4) TM Δβ > 0.2 for one histotype when compared to others (SI: Figure S2).

DMP analysis was performed for each possible histotype group comparison using ChAMP’s DMP function and minfi’s dmpFinder function using *M*-values, and probes were considered significant if they had adjusted *p*-value < 0.05 (BH) and further categorized as hypomethylated for their associated histotype group when β < 0.3, hemimethylated when 0.3 < β < 0.7 and hypermethylated when β > 0.7 [38]. Differentially methylated region (DMR) analysis was performed for each possible histotype group comparison using the DMRcate, probelasso, bumphunter (ChAMP) and sesame R packages (v.2.16.1) [39–42]. Regions consisting of *n* ≥ 3 CpGs with adjusted *p*-value < 0.05 and Δβ > 0.2 were deemed significant DMRs (SI: Table S1). To evaluate the frequency of promoter regions identified using the non-parametric approach (np-promoters) in the results from the aforementioned DMP and DMR callers, DMP and DMR CpG genomic coordinates were compared to np-promoter CpG site genomic coordinates using the GenomicRanges package (v.1.52.0) [43]. Lastly, for each promoter region of interest, the standard deviation (SD) and difference in TM were calculated between the training cohort TM and validation cohort TM for each CpG site.

### Predictive classification

CpG sites in np-promoters that passed the significance criterion in the training cohort, and who were present in both the EPIC and 450K arrays, were chosen as sets of CpGs representing their associated region. Gene-promoter CpG-sets were then used individually to create binary (one vs. many) XGBoost (XGB; xgboost v.1.7.8.1) [44] classifier models through using their CpG sets as predictors. Where the histotype group associated with thenp-promoter region was chosen as the case group and all other histotypes as the control group. Each such model was then subjected to 5-fold cross validation using stratified portioning of the training cohort data, repeated over 100 iterations, followed by the selection of the best model based on the average F1 score and precision recall (PR) area under the curve (AUC). The best model (i.e., set of CpGs used as predictors) was then chosen to represent that iteration of the sequential model selection, and the process was repeated a total of 5 times for each histotype. Meaning that the model for a histotype after the second iteration of the process, as an example, consisted of the best combination of the CpG set yielded in the first iteration coupled with the CpG set out of the remaining CpG sets for which it achieved the best predictive performance (SI: Figure S2-B). Repeating this process for each histotype and their associated np-promoter regions, the model with the best F1 score and PR AUC were chosen to represent their histotype. The best models for each histotype were then combined using grid search to obtain the best performing multiclass model based on PR-AUC.

This multiclass model was used to train an XGBoost classifier using the training cohort as training data and was then directly applied to a portion of the test-cohort datasets as test/holdout sets through splitting the test-cohort using 5-fold cross validation repeated over 100 iterations. Each histotype CpG model set was additionally used for binary classification in the test cohort datasets, through labeling samples associated with the histotype of interest as case, and all other samples as control, followed by direction application of the trained model on a 5-fold portion of the test-cohort dataset.

## Results

### CpG coverage and distribution types

Investigation of the CpG site coverage (*n* = 865859 CpG sites) for promoter regions in the EPIC array revealed that out of 20,033 protein coding genes retrieved from Ensembl, 18,944 genes had at least one CpG site overlapping the genomic coordinates of their promoter region. Out of these genes, 37.0% had *n* ≤ 6 CpG sites overlapping the genomic coordinates of their promoter region, 31% had 10 ≥ *n* ≥ 7 sites and 32% had *n* > 10 sites (Figure 1(A)). Naturally, this coverage was lower in the 450k array (*n* = 485512 CpG sites) with 52% of genes having *n* ≤ 6 overlapping CpG sites, and 7% of promoter regions with overlapping CpGs in the EPIC array lacking coverage in the 450K array. CpG’s in the training cohort (*n* = 736384 CpG sites) were found to have a similar number of hypo-, hemi-, and hypermethylated sites (Hypo: ~30%, Hemi: ~20%, Hyper: ~50%; SI: Figure S3) across histotypes, with the same pattern observed in the two test cohort datasets.

Despite the homogeneity observed for EOC histotype β-value methylation profiles, their associated distribution types varied considerably compared to each other, with many CpGs displaying non-normal distribution patterns for a histotype in the training cohort as well the test-cohort datasets (Train: 24.7–80.1%; Figure 1(B)). For *M*-values, a larger number of CpG sites adhered to a normal distribution pattern (54.8–86.2%), however, the fraction of CpG sites displaying non-normal distribution was still noticeable (13.8–45.2%). Even for adjacent CpGs within the same promoter region, the distribution type was shown to vary between groups, and within the group to a lesser degree (Figure 1(C)). Out of the histotypes, the smallest group (MC) had the highest frequency of normally distributed sites (75.3%), the lowest number of lightly skewed sites (20.3%), and no CpG sites exhibiting a heavy tail distribution. Inversely, the highest number of CpG sites following a skewed distribution was found for the largest group (HGSC; Light Skew: 27.5%, Heavy skew: 30.2%), which also exhibited the lowest number of normally distributed sites (20%). These patterns were further observed in the test cohorts, where CpG sites in HGSC samples, when compared to other histotypes, were found to adhere more frequently to a heavily skewed distribution (GSE226823; 23.7%, GSE51820; 18.9%) and had the lowest frequency of sites adhering to a normal distribution (GSE226823; 21.8%, GSE51820; 38.3%). CpG sites in MC samples instead showcased the highest number of CpG sites adhering to a normal distribution in GSE51820 (75.2%), with no sites adhering to a heavily skewed distribution. No discernable patterns were observed for CCC and EC, who instead showed a higher degree of heterogeneity between the training and test-cohort datasets.

### Differential promoter methylation analysis and differential methylation analysis

**Figure 2.**
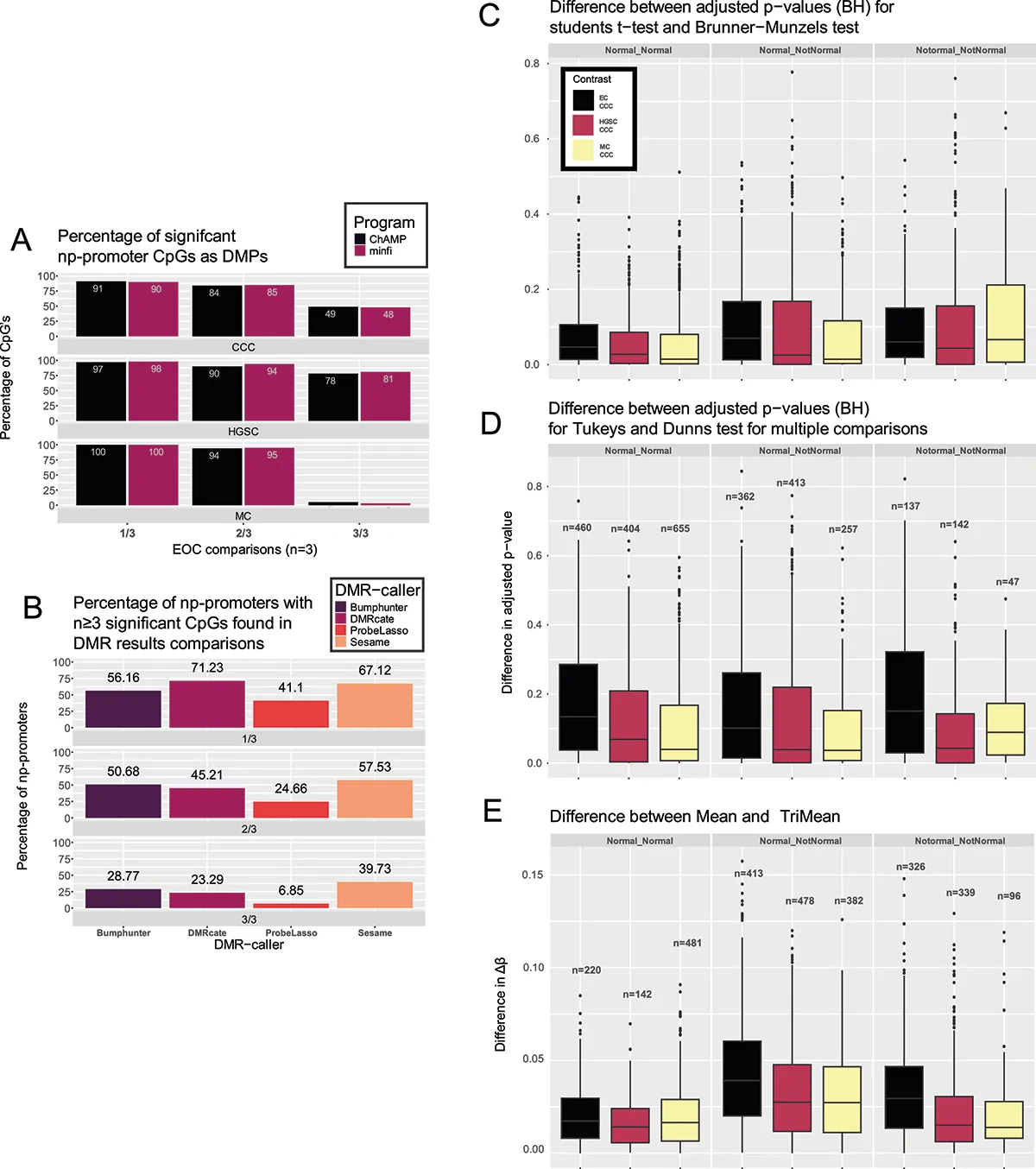
Comparison of np-promoter regions against results from DMP and DMR callers, as well as statistics for distribution types in comparisons (A) Comparison of significant np-promoter regions CpGs against minfi dmpFinder and Champ.DMPs (limma) results, (B) Comparison of np-promoter regions against other selected DMR callers. (C) Difference in Δβ for mean and TM in group comparisons using CpGs in all np-promoter regions, seen to the distribution types in the comparisons made (D) Difference in adjusted *p*-value (BH) between Brunner–Munzel and students t-test in comparisons using *M*-values for CpGs in all np-promoter regions, seen to the distribution types in the comparisons made, (E) Difference in adjusted *p*-value (BH) between Tukey’s test and Dunn’s test in comparisons using *M*-values for CpGs in all np-promoter regions, seen to the distribution types in the comparisons made. Np-promoter: Promoter region for a gene deemed uniquely significantly differentially methylated by the non-parametric approach described in the manuscript, EOC: Epithelial Ovarian Cancer, CCC: Clear Cell Carcinoma, EC: endometrioid carcinoma, HGSC: High Grade Serous Carcinoma, MC: Mucinous Carcinoma, TM: Tukeys Trimean, normal: Shapiro–Wilks test of normality *p* > 0.05, NotNormal: Shapiro–Wilks test of normality *p* < 0.05, DMP: Differentially methylated probe, DMR: Differentially methylated region, BH: Benjamini-Hochberg.

The non-parametric approach for promoter methylation resulted in 35 np-promoters for CCC, 0 for EC, 31 for HGSC, and 54 for MC (Total: 120; SI: Table S2-S4). CpG sites in np-promoters for CCC were generally hemimethylated in CCC and hypomethylated in other histotypes, CpG sites in np-promoters for HGSC were overall hypomethylated in HGSC compared to others and CpG sites in np-promoters for MC were overall hemimethylated in MC, but hypermethylated in others (SI: Table S5-S7). Comparison of CpG sites in np-promoters with ChAMP and Minfi DMP’s (SI: Figure S7–S8) revealed that most CpG’s fulfilling significance requirement in the promoter methylation analysis, were also deemed statistically significant DMPs in 1 or 2/3 comparisons (91–100%, 84–91% of CpGs found as DMPs; Figure 2(A)), but lower for DMPs found in 3/3 comparisons, except in comparisons with HGSC. Comparing np-promoter regions that contain *n* > 3 significant CpG sites to genomic coordinates of significant DMRs from bumphunter, DMRcate, probelasso and sesame revealed that between 6.9% and 39.7% (ProbeLasso, Sesame) of np-promoter regions were found to overlap genomic coordinates of DMRs with one or more CpG sites (Figure 2(B)). This number was found to be consistently higher when looking at overlaps in 1/3 and 2/3 possible comparisons (highest overlap: 71.23% DMRcate 1/3, 57.5% Sesame 2/3), indicating reproducibility with established methods but also the characterization of regions not covered by the other DMR callers. Comparison between the DMR caller results showed that the general reproducibility of DMR results in other callers for the same group comparison was consistently lower, with the exception of DMRs from sesame (SI: Figure S9–S10).

### Distribution types and impact on significance and effect size

Investigation of the DMPs deemed as significant in the non-parametric approach, but not in the DMP callers revealed that a majority of CpGs for which significance in a between-group comparison differed, were in comparisons where one or both groups did not adhere to the assumption of normality for all comparisons (SI: Figure S11–S12). Comparing statistical significance for CpG sites in np-promoter regions using parametric and non-parametric tests showed variation between the methods when using both M and β-values. Although no clear effects on significance could be contributed to the distribution types being compared for both between group comparisons (t-test and Brunner–Munzel; Figure 2(C)) and multiple pairwise comparisons (Tukeys test and Dunns test; Figure 2(D)). The mean variation between the parametric and non-parametric methods was often larger than 0.1 for the comparison between two groups, and the variation and relative effect of distribution types increased when utilizing the comparatively more heteroskedastic β-values (SI: Figure S13–S14). The comparison of TM on β-values showed a noticeably larger difference between the estimation for effect size when one group in a comparison did not adhere to the assumption of normality for all histotypes (Figure 2(E)).

### Predictive classification

**Figure 3.**
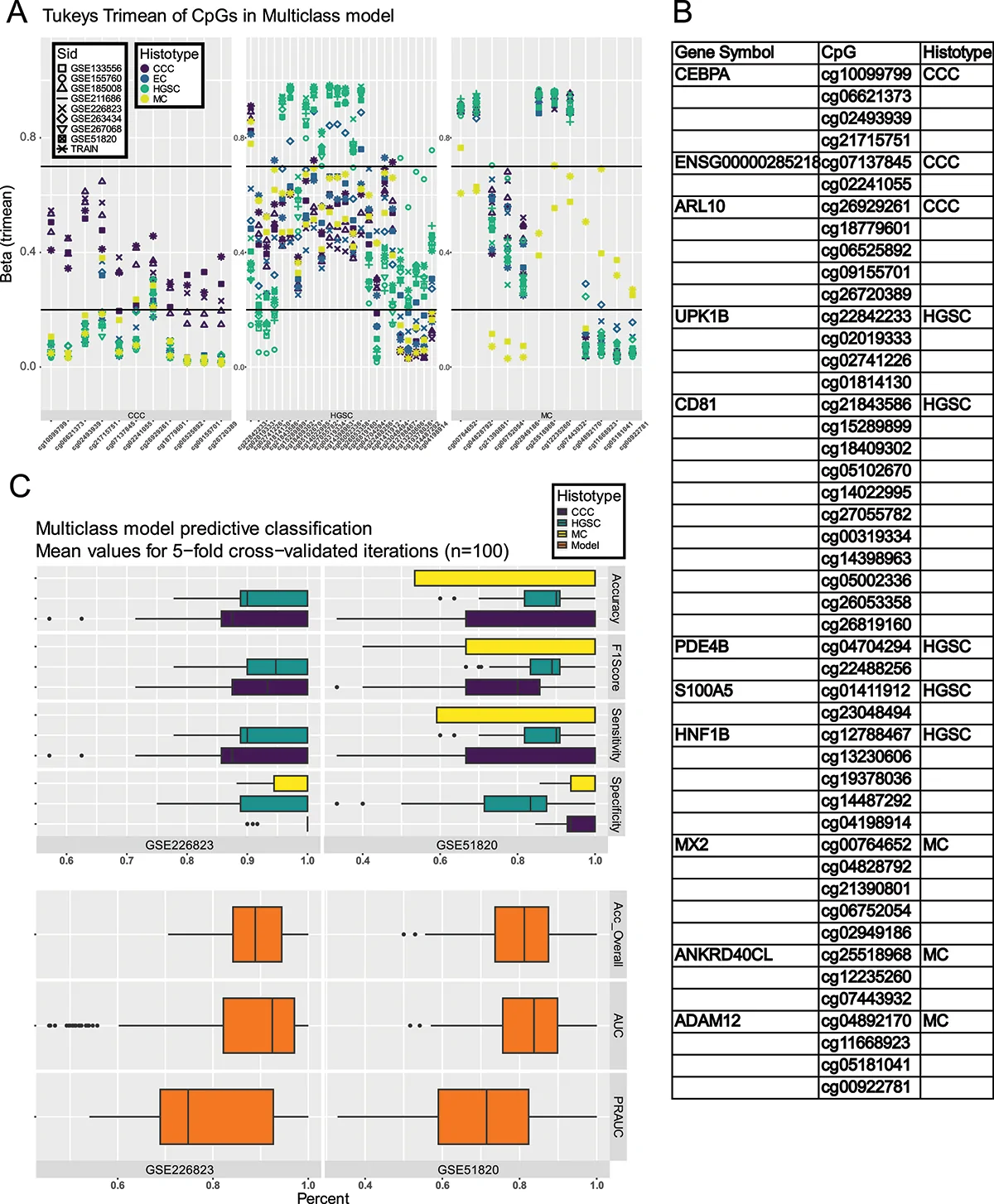
Statistics for multiclass classification of EOC histotypes using XGBoost in test-cohorts GSE51820 and GSE226823. Values in classification statistics are representative of the mean of 5-fold cross-validation of the test-cohorts, repeated over 100 iterations. (A) Trimean dot plot of multiclass model in cohorts. (B) Multiclass model showing CpGs in model, the histotype associated with the np-promoter region in which the CpG is located, and the HGNC symbol associated with the np-promoter region. (C) Predictive statistics for the multiclass model for individual histotypes (upper) and the model (lower). Np-promoter: Promoter region for a gene deemed uniquely significantly differentially methylated by the non-parametric approach described in the manuscript, CCC: Clear Cell Carcinoma, EC: Endometroid Carcinoma, HGSC: High-Grade Serous Carcinoma, MC: Mucinous Carcinoma, SID: Gene Expression Omnibus (GEO) id, AUC: Area Under Curve, PR: Precision recall, Range: Lower and upper bounds for multiclass model, OA: Overall Accuracy.

The stepwise selection process (SI: Figure S15) followed by grid search resulted in a multiclass model with 47 total features (CpGs) based on the promoter region of 11 genes (CCC: *CEBPA*, *ENSG00000285218*, *ARL10*, HGSC: *UPK1B*, *CD81*, *PDE4B*, *S100A5*, *HNF1B*, MC: *MX2*, *ANKRD40CL*, *ADAM12*); Figure 3(A,B)). When the classifier model that was trained on the training cohort data was directly applied to the test cohort datasets, AUC ranged between 82.6% and 88% and mean PR AUC between 70% and 79%. Histotypes CCC and HGSC showed sensitivity and F1 score 76–94%, while MC had a sensitivity and F1 score of ~60% in GSE51820. Specificity was found to be over 95% for all histotypes except for HGSC in GSE51820 (range: 81–98%; Figure 3(C); SI: Table S12–S13). Binary classification using CpGs from the multiclass model associated with the reference group as predictors showed similar performance to the multiclass model. Sensitivity was found to range between 77% and 93% for CCC and HGSC, with MC performing worse at 63%. Specificity was > 80% for all histotypes except HGSC in GSE51820 (72%), and AUC and PR AUC were found to be above 70% for all histotypes in both cohorts (SI: Figure S16, Table S14–S15).

### Literature review

Literature reviews of the np-promoter regions using OncoScore [45] showed that 20/35 CCC, 20/31 HGSC and 39/54 MC np-promoter region genes were significantly associated with cancer in existing literature. With genes such as *CYP2W1*, *LRCC41*, and *WT1* in CCC [46–48], *CD81*, *CMTM2* and *ZNF177* in HGSC [49–51] and *TFF1*, *TMEM220* and *ZNF471* in MC [52–54] having existing literature implicating their epigenetic involvement in cancer (SI: Table S16–S18).

### External validation

**Figure 4.**
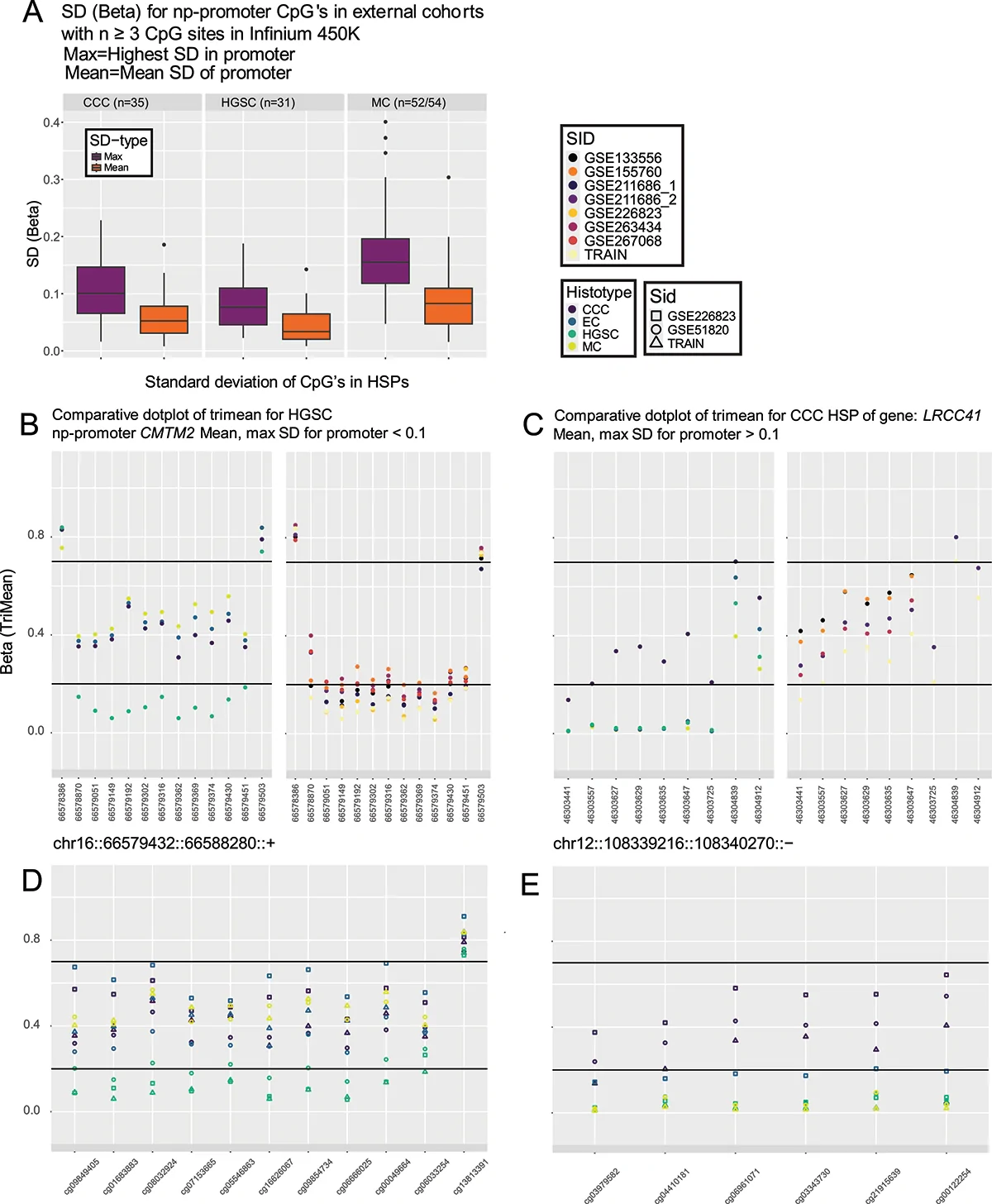
Np-promoter methylation pattern examples in training cohort and external cohorts. (A) Boxplots showing the β SD when compared to the training cohort TM β for all histotype np-promoters. (B) Example HGSC np-promoter CMTM2 with mean and max β SD below 0.1, left plot shows the trimean for all histotypes in training cohort. Right plot shows trimean for CCC samples in training and external cohorts (C) Example CCC np-promoter LRRC41 with mean and max β SD above 0.1, left plot shows the trimean for all histotypes in training cohort. Right plot shows trimean for CCC samples in training and external cohorts (D) Example HGSC np-promoter CMTM2, the plot shows the trimean for all histotypes in the training and test cohorts. (E) Example CCC np-promoter LRCC41, the plot shows the trimean for all histotypes in the training and test cohorts. Cpgs present in figure B, C but not in D, E explained by missingness in Infinium 450K arrays. Np-promoter: Promoter region for a gene deemed uniquely significantly differentially methylated by the non-parametric approach described in the manuscript, SD: Standard Deviation (based on training cohort vs. external cohorts), max SD: largest observed SD in the np-promoter region, mean SD: mean SD for the np-promoter region, CCC: clear cell carcinoma, EC: endometrioid carcinoma, HGSC: high-grade serous carcinoma, MC: mucinous carcinoma, Trimean: Tukeys Trimean, Hypo: Hypomethylated β < 0.3, Hemi: Hemimethylated: 0.3 < β < 0.7, Hyper: Hypermethylated 0.7 < β.

Comparing the TM values in the training cohort to the external cohorts (Figure 4(A)) revealed that out of the 35 CCC np-promoters, 17 were found to have max SD < 0.1 for any CpG site in the np-promoter regions and mean SD was found to be *n* < 0.1 for 33/35 promoter regions (Example SD < 0.1 Figure 4(B,D); Example SD > 0.1 Figure 4(C,E); smallest max SD = 0.02, *NUDT19*; SI: Table S19). Of the external HGSC datasets, 22/31 np-promoters had a max SD < 0.1 for any CpG within the regions and 29/31 regions had a mean SD < 0.1 (best max SD = 0.02, *WT1*: SI: Table S20). External SD calculations for MC were performed against only one dataset, as no other publicly available datasets with *n* > 5 MC samples originating from the 450K or EPIC platform were found at the time. A total of 8/52 promoter regions mapping to the 450K array had max SD < 0.1 for any CpG within the np-promoter (best max SD = 0.05, *ABCG5*; SI: Table S21). Applying the np-promoter approach on the test cohort datasets showed that 16/29 CCC and 16/29 np-promoters for HGSC with *n* > 3 CpGs in the 450K array (104/120 np-promoters with *n* > 3 CpGs) passed the filtering criterions in GSE226823 while 9/29 CCC, 1/29 HGSC, and 15/46 MC np-promoter regions were considered significant in GSE51820. Further inspection of the results showed that np-promoters for HGSC generally passed the significance thresholds when compared to CCC, but failed significance thresholds when compared against EC for which it showed higher degrees of homogeneity, and to a lesser degree in comparisons with MC.

## Discussion

As the aim of this study was to investigate distribution types in EOC methylation data and their influence on effect size measurements and statistical significance, we implemented non-parametric statistical tests and a robust effect size method to characterize pre-defined promoter regions that showed significant differential methylation. Results indicate that that the distribution type of groups being compared influence the statistical outcome when measuring effect size based on the use of parametric and non-parametric methods, that parametric and non-parametric approaches differ from one another even when applied to *M*-values, and lastly that the distribution types for EOC methylation data differ between histotypes. Several of the characterized promoter regions, such as those corresponding to *LRRC41* and *PYY* for CCC, *CD81, CMTM2*, and *TGM1*for HGSC and *BATF*, *LGALS4* and *ZSCAN1* for MC, were found to have highly reproducible uniquely methylated differentiated patterns for one histotype across cohorts, and a CpG panel created from such np-promoter regions was able to stratify histotypes from each other in both multiclass and binary classification models applied to external datasets.

The results from the distribution-type analysis show that CpG site DNA methylation β-values, where one methylation state (hypo or hypermethylated) tend to be more prevalent [55], often follow a skewed or non-normal distribution in EOC histotypes, with a lesser but still noticeable presence in *M*-values. Additionally, HGSC and MC seem to exhibit distribution type patterns in a way that is reproducible across cohorts. With HGSC having a higher frequency of CpG sites with heavily skewed distributions, and MC a higher frequency of normally distributed CpG sites, holding true even for the more homoscedastic *M*-values. It is debatable whether the differences observed are due to within-group heterogeneity or due to histotype-specific methylation patterns. Especially in the case of the MC histotype for which only a low number of samples were available in both the training cohort and external cohorts. As the influence of outliers is more influential in smaller groups, findings related to the MC groups could be skewed statistically and should be evaluated in cohorts with larger sample sizes. However, the fact remains that for all histotypes investigated in this study, many CpG sites do not have methylation values that adhere to the assumption of normality.

Considering these findings, methods that assume that groups who are being compared adhere to this assumption such as the 2-sided t-test of F-statistic, may be suboptimal for use in DNA methylation data, as the statistical outcome could be influenced by the distribution type(s) of the data of the groups being compared [56]. Similarly, methods sensitive to outliers such as the arithmetic mean are a poor choice for calculating effect-sizes in DNA-methylation data, as the measure of central tendency will be impacted if it does not adhere to the assumption of normality [57,58]. Whereas methods that ignore the tail ends of a distribution such as the median instead risk masking true biological variability for the sake of robustness. Comparing the arithmetic mean to TM for β-values, and the students t-test to Dunn’s test for *M*-values (SI: Figure S4-S10) reinforced the assumption of the distribution type of data in comparisons influencing results. As the highest disagreement between the parametric and non-parametric methods was found in comparisons where at least one group did not adhere to the assumption of normality.

The same pattern was further observed when investigating differences between the DMPs characterized using the non-parametric approach and the DMPs characterized by the two chosen DMP-callers. Where a majority of disagreements regarding the significance of a CpG site were observed in comparisons involving at least one distribution not adhering to the assumption of normality (Figure 2D; SI: Figure S11). Inversely, the most common comparison type for CpGs deemed significant by both the DMP caller and the non-parametric approach were between two normal distributions. Seeing how the mean *p*-value difference between the normal-normal distribution type comparisons, when compared to other comparison types, was often larger than 0.05. It stands to reason that programs utilizing methods which handle non-normally distributed data poorly may yield false positives or negatives through over or underestimation of the statistical significance of a CpG site due to the distribution types of the groups being compared. From a biological perspective, this may pose a problem in studies involving groups with high within group heterogeneity. Or for studies where the availability of data is limited (such as for studies involving rarer disease types), as the effects on outliers will be more pronounced in groups with smaller sample sizes.

It should be stated that the non-parametric approach used to detect differentially methylated regions in this study is a simplistic approach that does not consider factors such as regional homogeneity of methylation or the spatial correlation of CpG sites within or adjacent to the promoter region. Furthermore, the cutoff threshold Δβ > 0.2 (equivalent to a ~ 20% difference in methylation between two groups being compared) used in this study was chosen based on common convention rather than a statistical basis, since what should be considered an optimal cutoff threshold for methylation differences between groups is highly contextual based on the goal of the study. The methodology discussed was further hindered by the uneven CpG coverage observed in the Infinium EPIC and 450K arrays, where nearly X% and Y% of all gene promoter regions were not eligible for analysis due to containing less than three overlapping CpG sites, respectively. Similarly, regions with adequate but poor CpG coverage could have been deemed statistically insignificant due to overlapping significant CpG sites in the EPIC array missing in the 450K array during downstream analysis.

Data in the external cohorts likely influenced results through sources of variability through factors such as protocols used when generating the data, technical platform, geographical origin of the patients and other external factors [59–62]. Additionally, datasets in this study used different guidelines for histotype classification based on the FIGO (International Federation of Gynecology and Obstetrics) recommendations from their year of origin, meaning that histotype classification may not be consistent across the datasets. Finally, the small and unbalanced sample sizes in the test and training cohorts, especially for the rarer histotypes such as MC, likely influenced results through inflated *p*-values, overfitting and the presence of outliers. Despite these limitations, np-promoter regions showed a high degree of reproducibility with low SD across cohorts for the chosen histotype. Being reproducible in results from established DMP and DMR callers, while also identifying regions of significance missed by other approaches.

Classification models originating from CpGs in np-promoters that were directly applied on external data without batch correction, after being trained on the training cohort, successfully stratified most histotypes across the test-cohort datasets in both binary and multiclass models. Strengthening the idea of the np-promoters as candidate biomarkers for the purpose of histotype stratification in methylation data. It is likely that the performance could be further improved if applied on a full set of np-promoter CpGs in the EPIC array, as CpG sites, which underwent feature selection were limited to those shared across the EPIC and 450K array after quality control (*n* = 366518 CpG sites). Meaning that many potential predictors were ignored when creating the model due to missingness in the 450K arrays. And as such further validation in external data is needed to ensure that the observed predictive performance is reproducible, especially for the smaller MC group.

Unlike what was seen for the other histotypes in the study, no np-promoter regions were characterized in the training cohort for the EC histotype, a phenomenon that was observed to stem from homogeneity with the HGSC or the MC histotypes, respectively. Candidate regions that displayed heterogeneity for EC in 2/3 comparisons, failed in comparison with either the HGSC or MC histotype, a phenomenon that was also seen through the low number of DMPs and DMRs. This dualistic nature could possibly be explained by differences between low- and high-grade EC, as all cohorts included in the study were of mixed or unspecified grades. Where low-grade EC is known to more closely resemble non-serous histotypes such as MC and CCC, whereas high-grade EC instead is known to more closely resemble serous histotypes such as HGSC [63]. Although the proposed np promoter-regions for CCC, HGSC and MC differ significantly from the EC histotype, further investigation should ideally be made to evaluate if the EC histotype should be considered as dual entity in the form of low- and high-grade EC when attempting to characterize epigenetic signatures, to better reflect its dualistic clinical behavior.

Although these findings indicate that the distribution type of compared sums do influence the statistical outcome of differential methylation analysis based on the use of parametric and non-parametric methods and characterized several gene promoter regions showing aberrant methylation uniquely associated with one histotype in EOC. Further, statistical evaluation is needed before any conclusions can be drawn regarding the performance of the approaches used in this study in relation to existing methods for DMP and DMR analysis. It is likely that more regions of interest could be identified through a generalist implementation of the presented methodology beyond pre-defined gene promoter regions (such as regression or sliding-window-based approaches), using parameters for significance determined through rigorous statistical evaluation on larger datasets in a non-explorative analysis. Or when applied to data originating from methods providing better coverage such as whole-genome bisulphite sequencing. However, such assumptions need to be explored thoroughly before warranting further discussion. Lastly, all findings presented in this study are based on the training cohort, which consists of data originating from solid tissue samples without paired normal tissue. As such, the relation of the presented findings to that of normal tissue, as well as their applicability in circulating tumor DNA (ctDNA) has yet to be confirmed and needs to be investigated before any conclusions regarding their applicability for diagnostic use can be drawn.

## Conclusion

In this study, we utilized non-parametric methods for statistical significance and effect size estimation to characterize promoters uniquely aberrantly methylated for three of the four major EOC histotypes. Results show that EOC histotypes are epigenetically heterogenous, and that EOC methylation does not adhere to the assumption of normality. With the methylation patterns observed in CpG sites adhering to half-normal or skewed distributions for both β, and to a lesser extent *M*-values. These distribution types were further shown to impact the statistical outcome of the analysis as parametric and non-parametric results varied for both β and *M*-values, with the results of effect size estimation using mean and TM for β values diverging more strongly when one or both compared sums did not adhere to the assumption of normality. Raising the importance of choosing suitable methods for the data being analyzed when attempting to detect aberrant methylation between groups, especially when making cutoffs based on β values. The gene promoter regions identified through the non-parametric approach were shown to be uniquely methylated for one histotype, reproducible in established methods for DMP and DMR detection, correlated with cancer in existing literature, and to be highly reproducible in external datasets. And classification models constructed from CpGs in such regions yielded PR AUC scores > 70% for all histotypes except EC. While the low number of datasets with a majority of EOC histotypes represented used in the study, and oftentimes low sample sizes for a histotype must be acknowledged as limitations. The presented np-promoter regions show promise as prospective candidate biomarkers for histotype stratification in EOC DNA-methylation data originating from sample tissue.

## Supplementary Material

hugo_swenson_revision_Supplementary_Information_clean.docx

## Data Availability

Code utilized in the creation of this manuscript can be retrieved through the GitHub repository: https://github.com/ehugos/NonParam-Promoters-EOC All data used in the study are available under their corresponding GEO accession codes. Training cohort data as an example is available at GEO under accession code GSE326000. GSE326000: https://www.ncbi.nlm.nih.gov/geo/query/acc.cgi?acc=GSE326000 GSE51820: https://www.ncbi.nlm.nih.gov/geo/query/acc.cgi?acc=GSE51820 GSE226823: https://www.ncbi.nlm.nih.gov/geo/query/acc.cgi?acc=GSE226823 GSE133556: https://www.ncbi.nlm.nih.gov/geo/query/acc.cgi?acc=GSE133556 GSE211686 (450k (1) and EPIC (2): https://www.ncbi.nlm.nih.gov/geo/query/acc.cgi?acc=GSE211686 GSE263434: https://www.ncbi.nlm.nih.gov/geo/query/acc.cgi?acc=GSE263434 GSE267068: https://www.ncbi.nlm.nih.gov/geo/query/acc.cgi?acc=GSE267068 GSE155760: https://www.ncbi.nlm.nih.gov/geo/query/acc.cgi?acc=GSE155760 GSE185008 (450k (1) and EPIC (2): https://www.ncbi.nlm.nih.gov/geo/query/acc.cgi?acc=GSE185008
