# Supplementary material for: Non-parametric differential methylation analysis characterizes histotype-specific promoter regions in epithelial ovarian cancer
Source: Epigenetics. 2026 Sep 2;21(1):2720990. doi: 10.1080/15592294.2026.2720990 (PMC13540201; doi:10.1080/15592294.2026.2720990)
Supplement: Supplementary file 1 [file KEPI_A_2720990_SM3138.docx]

**Supplementary Information (SI)**

**Article title**

Differential methylation of promoters as a tool for histotype stratification in epithelial ovarian cancer

**Journal**

Epigenetics (Taylor & Francis)

**Authors**

Hugo Swenson, Ella Ittner, Lucas Werner, Elisabeth Werner Rönnerman, Szilárd Nemes, Toshima Z. Parris, and Khalil Helou

Corresponding author

Hugo Swenson, [hugo.swenson@gu.se](mailto:hugo.swenson@gu.se)

**Figure 1:** PCA and density plots of Training and Test cohorts

*Principal Component Analysis (PCA) plots of the training and test cohort top 1000 most variable probes in the array, and corresponding β-value density plots for all CpG sites investigated. (A) Training cohort, (B) GSE51820, (C) GSE226823. CCC: Clear Cell Carcinoma, EC: Endometroid Carcinoma, HGSC: High Grade Serous Carcinoma, MC: Mucinous Carcinoma*

**Figure 2:** Flowcharts of NP-PROMOTER methodology and sequential NP-PROMOTER model selection for predictive classification

*Flowchart for the np-promoter methodology, TM: Tukeys Trimean, BH: Benjamini Hochberg, NP-Promoter: Uniquely differentially methylated promoter region characterized by the non-parametric methodology in manuscript*

**Table 1:** DMP, DMR packages and parameters used

| **Package** | **Type** | **Version** | **Parameters** | **Additional filtering** |
| --- | --- | --- | --- | --- |
| minfi | DMP | 1.48.0 | type = "categorical"  qCutoff = 0.05 |  |
| ChAMP | DMP | 2.32.0 | adjPVal = 0.05  adjust.method = "BH" |  |
| ChAMP (bumphunter) | DMR | 2.32.0 | method = “Bumphunter",  minProbes = 3  adjPvalDmr=0.05  B=100  pickCutoff = 0.95 | n ≥2 CpGs |
| DMRcate | DMR | 2.16.1 | lambda=1000  C=2  betacutoff=0.2  min.cpg=3 | n ≥3 CpGs  Adjusted P < 0.05 |
| ProbeLasso | DMR | 2.20.0 | adjPvalProbe = 0.05 | n≥3CpGs |
| Sesame | DMR | 1.20.0 | Platform = EPIC,  seg.per.locus = 0.5 | 3 ≥2 CpGs  Seg_Pval_adj < 0.05 |

*Packages used in DMP, DMR analysis alongside parameters used*

**Figure 3:** Beta value methylation types in the training cohort

*Methylation type for all samples and CpG sites in the training cohort. Percent refers to the number of CpG sites for all samples adhering to a methylation type, divided by the number of CpG sites tested (n=736384) times the number of samples belonging to the histotype in question. Hypo: β < 0.3, Hemi: 0.3 < β < 0.7, Hyper: β > 0.7, CCC: Clear Cell Carcinoma, EC: Endometroid Carcinoma, HGSC: High Grade Serous Carcinoma, MC: Mucinous Carcinoma*

| **ensembl_gene_id** | **external_gene_name** | | | | | | | | | | | | **TM_Diff_EC** | | | | | | | | | | | | **TM_Diff_HGSC** | | | | | | | | | | | | | | | | | | | | | | | **TM_Diff_MC** | **nCpG** | **sigCpG** |
| --- | --- | --- | --- | --- | --- | --- | --- | --- | --- | --- | --- | --- | --- | --- | --- | --- | --- | --- | --- | --- | --- | --- | --- | --- | --- | --- | --- | --- | --- | --- | --- | --- | --- | --- | --- | --- | --- | --- | --- | --- | --- | --- | --- | --- | --- | --- | --- | --- | --- | --- |
| ENSG00000006025 | | | | | | OSBPL7 | | | | | | | | | 0.228370508833134 | | | | | | | | | 0.225165541099774 | | | | | | | | | | 0.280006501083213 | | | | | 3 | | | | | | | 3 | |  |  |  |
| ENSG00000135392 | DNAJC14 | | | | | | | | | | | | 0.216563340952342 | | | | | | | | | | | | 0.233889797343345 | | | | | | | | | | | | | | | | | | | | | | | 0.243473904840617 | 6 | 4 |
| ENSG00000139914 | FITM1 | | | | | | | | | | | | 0.251432402217437 | | | | | | | | | | | | 0.242803105091997 | | | | | | | | | | | | | | | | | | | | | | | 0.268879678072098 | 4 | 2 |
| ENSG00000184956 | MUC6 | | | | | | | | | | | | 0.106898117740045 | | | | | | | | | | | | 0.13917736392725 | | | | | | | | | | | | | | | | | | | | | | | 0.250492893087695 | 11 | 5 |
| ENSG00000106384 | MOGAT3 | | | | | | | | | | | | 0.127067371743557 | | | | | | | | | | | | 0.174398888808822 | | | | | | | | | | | | | | | | | | | | | | | 0.244021081605843 | 12 | 5 |
| ENSG00000132128 | LRRC41 | | | | | | | | | | | | 0.211848237489022 | | | | | | | | | | | | 0.241657467018496 | | | | | | | | | | | | | | | | | | | | | | | 0.275277252416924 | 9 | 3 |
| ENSG00000168439 | STIP1 | | | | | | | | | | | | 0.190950301470068 | | | | | | | | | | | | 0.159856272804382 | | | | | | | | | | | | | | | | | | | | | | | 0.194110756087535 | 8 | 2 |
| ENSG00000213965 | NUDT19 | | | | | | | | | | | | 0.055086661722041 | | | | | | | | | | | | 0.0368676278228381 | | | | | | | | | | | | | | | | | | | | | | | 0.0947267033705752 | 8 | 2 |
| ENSG00000175414 | | ARL10 | | | | | | | | 0.231567769279785 | | | | | | | 0.21852953762525 | | | | | | | | | 0.226591967326751 | | | | | | | | | 12 | | | | | | | | | 6 | | | |  |  |  |
| ENSG00000245848 | | CEBPA | | | | | | | | 0.244089527102949 | | | | | | | 0.249333861258448 | | | | | | | | | 0.2354013226264 | | | | | | | | | 6 | | | | | | | | | 4 | | | |  |  |  |
| ENSG00000073712 | | FERMT2 | | | | | | | | 0.149555426675721 | | | | | | | 0.137938999361216 | | | | | | | | | 0.177203131872931 | | | | | | | | | 6 | | | | | | | | | 2 | | | |  |  |  |
| ENSG00000161653 | | NAGS | | | | | | | | 0.195498605145862 | | | | | | | 0.267782706938008 | | | | | | | | | 0.261592229737122 | | | | | | | | | 12 | | | | | | | | | 5 | | | |  |  |  |
| ENSG00000237172 | B3GNT9 | | | | | | | | | | | | 0.23264015527363 | | | | | | | | | | | | 0.180455176250335 | | | | | | | | | | | | | | | | | | | | | | | 0.236738998150928 | 7 | 3 |
| ENSG00000187554 | TLR5 | | | | | | | | | | | | 0.113363543429657 | | | | | | | | | | | | 0.124330568462059 | | | | | | | | | | | | | | | | | | | | | | | 0.175862429791546 | 10 | 3 |
| ENSG00000183560 | | IZUMO1R | | | | | | | | | | 0.234096820237604 | | | | | | | | 0.225449801722456 | | | | | | | | 0.247325986428717 | | | | | | | | | | 5 | | | | | | 2 | | | |  |  |  |
| ENSG00000167701 | GPT | | | | | | | | | | | | 0.329413126768206 | | | | | | | | | | | | 0.418644932140925 | | | | | | | | | | | | | | | | | | | | | | | 0.349464292422399 | 4 | 2 |
| ENSG00000124818 | OPN5 | | | | | | | | | | | | 0.100594712919902 | | | | | | | | | | | | 0.110730390917347 | | | | | | | | | | | | | | | | | | | | | | | 0.112565614994364 | 8 | 3 |
| ENSG00000249481 | SPATS1 | | | | | | | | | | | | 0.224913382731563 | | | | | | | | | | | | 0.18579072746702 | | | | | | | | | | | | | | | | | | | | | | | 0.23418869848983 | 9 | 3 |
| ENSG00000106336 | | | FBXO24 | | | | | | | | 0.244166323781356 | | | | | | | | 0.144098460587664 | | | | | | | | 0.239739429991367 | | | | | | | | | | 8 | | | | | | | 2 | | | |  |  |  |
| ENSG00000187800 | PEAR1 | | | | | | | | | | | | 0.110616721266621 | | | | | | | | | | | | 0.216529938448465 | | | | | | | | | | | | | | | | | | | | | | | 0.164597689417645 | 10 | 3 |
| ENSG00000181409 | | | | | | | AATK | | | | | | | | | 0.15273845792381 | | | | | | | 0.174091051548273 | | | | | | | | | | 0.142786921843804 | | | | | | 5 | | | | | | | 2 | |  |  |  |
| ENSG00000174600 | CMKLR1 | | | | | | | | | | | | 0.228874217224833 | | | | | | | | | | | | 0.295849165535234 | | | | | | | | | | | | | | | | | | | | | | | 0.319142038527769 | 10 | 2 |
| ENSG00000100253 | MIOX | | | | | | | | | | | | 0.215945807821317 | | | | | | | | | | | | 0.223130708790549 | | | | | | | | | | | | | | | | | | | | | | | 0.178609033702177 | 11 | 2 |
| ENSG00000183773 | | | | | | AIFM3 | | | | | | | | | 0.218958965425358 | | | | | | | | | 0.235834877211028 | | | | | | | | 0.223140714642218 | | | | 7 | | | | | | | 3 | | | | |  |  |  |
| ENSG00000269404 | SPIB | | | | | | | | | | | | 0.110895116678926 | | | | | | | | | | | | 0.127318225799396 | | | | | | | | | | | | | | | | | | | | | | | 0.138241169933873 | 7 | 3 |
| ENSG00000285218 | | | | | | | |  | | | | | | 0.311132755837074 | | | | | | | | 0.315744032308349 | | | | | | | | | 0.247746453037898 | | | | | | | | | 8 | | | | | 3 | | |  |  |  |
| ENSG00000013297 | CLDN11 | | | | | | | | | | | | 0.311132755837074 | | | | | | | | | | | | 0.315744032308349 | | | | | | | | | | | | | | | | | | | | | | | 0.247746453037898 | 8 | 2 |
| ENSG00000174326 | SLC16A11 | | | | | | | | | | | | 0.326664110901624 | | | | | | | | | | | | 0.319880972984723 | | | | | | | | | | | | | | | | | | | | | | | 0.29795549885612 | 7 | 2 |
| ENSG00000131096 | | PYY | | | | | | | | | 0.151646316752002 | | | | | | | 0.312185539273951 | | | | | | | | 0.286460339186144 | | | | | | | | | 16 | | | | | | | | | 2 | | | |  |  |  |
| ENSG00000073067 | | CYP2W1 | | | | | | | | | 0.209605576513429 | | | | | | | 0.2765940869588 | | | | | | | | 0.201045477645613 | | | | | | | | | 9 | | | | | | | | | 2 | | | |  |  |  |
| ENSG00000171124 | FUT3 | | | | | | | | | | | | 0.16621209626775 | | | | | | | | | | | | 0.186721514904844 | | | | | | | | | | | | | | | | | | | | | | | 0.19727618331361 | 8 | 2 |
| ENSG00000128567 | PODXL | | | | | | | | | | | | 0.135317034245174 | | | | | | | | | | | | 0.131051818353554 | | | | | | | | | | | | | | | | | | | | | | | 0.131427923278405 | 7 |  |
| ENSG00000110696 | | | | C11orf58 | | | | | | | | 0.0629233799133327 | | | | | | | | | 0.10983651155827 | | | | | | | | | 0.117102398279437 | | | | | | | | | | | | 7 | | | | |  |  |  |  |
| ENSG00000139154 | AEBP2 | | | | | | | | | | | | 0.209070089063618 | | | | | | | | | | | | 0.209066295252907 | | | | | | | | | | | | | | | | | | | | | | | 0.201289592289731 | 5 |  |
| ENSG00000075651 | | | | | PLD1 | | | | 0.209567034197949 | | | | | | | | | | | 0.16830792704603 | | | | | | | | | 0.143036975773548 | | | | | | | | | | | | 7 | | | | | |  |  |  |  |

*Distribution types for M and β-values in the training cohort. Normal: Shapiro-Wilks p > 0.05, NotNormal: Shapiro-Wilks p < 0.05, LightSkew: 1 < Skewness < 2, HeavySkew: Skewness > 2, CCC: Clear Cell Carcinoma, EC: Endometroid Carcinoma, HGSC: High Grade Serous Carcinoma, MC: Mucinous Carcinoma*

**Table 2:** Np-promoters’s for CCC

*Np-promoters for CCC. All np-promoters have n > 33% CpGs with Δ β > 0.2 and adjusted p < 0.05 and aggregated p-value of p<0.05 for all three histotype comparisons. Tm_Diff: The TM of the region, based on the TM of differences between the NP-PROMOTER group and the column histotype for each individual CpG site. NP-Promoter: Uniquely differentially methylated promoter region characterized by the non-parametric methodology in manuscript, nCpG: Number of CpG sites found overlapping with the genomic coordinates of the NP-PROMOTER region, sigCpG: Number of CpG sites deened statistically and biologically significant within the NP-PROMOTER region, CCC: Clear Cell Carcinoma, EC: Endometroid Carcinoma, HGSC: High Grade Serous Carcinoma, MC: Mucinous Carcinoma, TM: Tukeys Trimean*

**Table 3:** Np-promoters’s for HGSC

| **ensembl_gene_id** | **external_gene_name** | | | **TM_Diff_CCC** | | | | | | | **TM_Diff_EC** | | | | | | **TM_Diff_MC** | | | | | | | | **nCpG** | | | | | | | | **sigCpG** | | | |
| --- | --- | --- | --- | --- | --- | --- | --- | --- | --- | --- | --- | --- | --- | --- | --- | --- | --- | --- | --- | --- | --- | --- | --- | --- | --- | --- | --- | --- | --- | --- | --- | --- | --- | --- | --- | --- |
| ENSG00000108018 | SORCS1 | | | 0.239741086672439 | | | | | | | 0.154557671793411 | | | | | | 0.239860643710197 | | | | | | | | 12 | | | | | | | | 4 | | | |
| ENSG00000184937 | WT1 | | | 0.161513775098094 | | | | | | | 0.150382684767841 | | | | | | 0.146516708275885 | | | | | | | | 12 | | | | | | | | 4 | | | |
| ENSG00000145864 | GABRB2 | | | 0.156489103819405 | | | | | | | 0.162256029614478 | | | | | | 0.127725469000196 | | | | | | | | 8 | | | | | | | | 2 | | | |
| ENSG00000114638 | UPK1B | | | 0.274682273400905 | | | | | | | 0.366787364952408 | | | | | | 0.364108965906126 | | | | | | | | 7 | | | | | | | | 5 | | | |
| ENSG00000110651 | CD81 | | | 0.336326025323955 | | | | | | | 0.368309815768121 | | | | | | 0.296962862155341 | | | | | | | | 12 | | | | | | | | 11 | | | |
| ENSG00000140932 | CMTM2 | | | 0.280067302005184 | | | | | | | 0.309212829437646 | | | | | | 0.34980045398251 | | | | | | | | 13 | | | | | | | | 10 | | | |
| ENSG00000198626 | RYR2 | | | 0.200843638826821 | | | | | | | 0.255219667098874 | | | | | | 0.392935368310907 | | | | | | | | 6 | | | | | | | | 3 | | | |
| ENSG00000158050 | | | | | DUSP2 | | | | | | | 0.230088956144558 | | | | | | 0.168582081944521 | | | | | 0.228212915209607 | | | | | 11 | | | | | | | 5 | |
| ENSG00000092295 | TGM1 | | | 0.343310390497793 | | | | | | | 0.306058819251452 | | | | | | 0.315087426729098 | | | | | | | | 5 | | | | | | | | 4 | | | |
| ENSG00000144837 | PLA1A | | | 0.284131287956751 | | | | | | | 0.144246962632264 | | | | | | 0.192750615353167 | | | | | | | | 7 | | | | | | | | 3 | | | |
| ENSG00000124721 | DNAH8 | | | 0.17414531680945 | | | | | | | 0.139480572621716 | | | | | | 0.215032472482856 | | | | | | | | 8 | | | | | | | | 2 | | | |
| ENSG00000144407 | PTH2R | | | 0.279248920048577 | | | | | | | 0.234750381367022 | | | | | | 0.297245504474753 | | | | | | | | 5 | | | | | | | | 4 | | | |
| ENSG00000180878 | | | | | | | C11orf42 | | | | | | | 0.229922814050419 | | | | | | 0.241953479017267 | | | | | 0.201173826551345 | | | | | | 6 | | | | | 3 |
| ENSG00000275410 | HNF1B | | | 0.238954205773758 | | | | | | | 0.206381184397474 | | | | | | 0.241787892688146 | | | | | | | | 11 | | | | | | | | 6 | | | |
| ENSG00000204396 | VWA7 | | | 0.196255273638098 | | | | | | | 0.165128658182395 | | | | | | 0.181202013630116 | | | | | | | | 21 | | | | | | | | 8 | | | |
| ENSG00000184588 | | | PDE4B | | | | | | 0.161991870289888 | | | | | | | 0.116590489931059 | | | | | | 0.240705276077871 | | | | | | | 9 | | | | | 3 | | |
| ENSG00000172995 | ARPP21 | | | 0.231568682205221 | | | | | | | 0.242205430819573 | | | | | | 0.303301400529137 | | | | | | | | 6 | | | | | | | | 2 | | | |
| ENSG00000196420 | S100A5 | | | 0.250515203817321 | | | | | | | 0.24327460331958 | | | | | | 0.2531623896042 | | | | | | | | 4 | | | | | | | | 2 | | | |
| ENSG00000259900 |  | | | 0.13634092202813 | | | | | | | 0.179802287444757 | | | | | | 0.289224808706611 | | | | | | | | 8 | | | | | | | | 2 | | | |
| ENSG00000157315 | TMED6 | | | 0.13634092202813 | | | | | | | 0.179802287444757 | | | | | | 0.289224808706611 | | | | | | | | 8 | | | | | | | | 2 | | | |
| ENSG00000173212 | MAB21L3 | | | 0.354819797942388 | | | | | | | 0.251009891788846 | | | | | | 0.328155187442811 | | | | | | | | 3 | | | | | | | | 2 | | | |
| ENSG00000124782 | | | | | | RREB1 | | | | | 0.0536809006075968 | | | | | | | | 0.0568317420674115 | | | | | | | 0.0953227439373879 | | | | | | 9 | | | | 3 |
| ENSG00000127903 | | | | | | | | ZNF835 | | | | | 0.24856202875609 | | | | | 0.230881802653341 | | | | | | 0.415137482356558 | | | | | | 8 | | | | | | 3 |
| ENSG00000103569 | AQP9 | | | 0.178567398596551 | | | | | | | 0.189514221939323 | | | | | | 0.173030621960587 | | | | | | | | 7 | | | | | | | | 3 | | | |
| ENSG00000125533 | BHLHE23 | | | 0.143927389229155 | | | | | | | 0.114526268637353 | | | | | | 0.205361904982461 | | | | | | | | 10 | | | | | | | | 3 | | | |
| ENSG00000188629 | ZNF177 | | | 0.182490726547588 | | | | | | | 0.162299210910418 | | | | | | 0.142539188226524 | | | | | | | | 10 | | | | | | | | 3 | | | |
| ENSG00000110079 | MS4A4A | | | 0.289454037866999 | | | | | | | 0.196956240824596 | | | | | | 0.339145280854288 | | | | | | | | 7 | | | | | | | | 2 | | | |
| ENSG00000206530 | | CFAP44 | | | | | | | | 0.204233550990118 | | | | | 0.212350152960321 | | | | | | 0.184387387227425 | | | | | | 12 | | | | | | | | 4 | |
| ENSG00000151365 | THRSP | | | 0.166961790338389 | | | | | | | 0.122382430264993 | | | | | | 0.149642890762706 | | | | | | | | 5 | | | | | | | | 2 | | | |
| ENSG00000197915 | HRNR | | | 0.164880015283882 | | | | | | | 0.143841431028436 | | | | | | 0.19923540625822 | | | | | | | | 4 | | | | | | | | 2 | | | |
| ENSG00000129467 | ADCY4 | | | 0.113449923836251 | | | | | | | 0.0775057071267202 | | | | | | 0.0971754115708293 | | | | | | | | 6 | | | | | | | | 2 | | | |

*NP-PROMOTERs for HGSC. All NP-PROMOTERs have n > 33% CpGs with Δ β > 0.2 and adjusted p < 0.05 and aggregated p-value of p<0.05 for all three histotype comparisons. Tm_Diff: The TM of the NP-PROMOTER region, based on the TM of differences between the NP-PROMOTER group and the column histotype for each individual CpG site. NP-PROMOTER: Histotype specific promoter, nCpG: Number of CpG sites found overlapping with the genomic coordinates of the NP-PROMOTER region, sigCpG: Number of CpG sites deened statistically and biologically significant within the NP-PROMOTER region, CCC: Clear Cell Carcinoma, EC: Endometroid Carcinoma, HGSC: High Grade Serous Carcinoma, MC: Mucinous Carcinoma, TM: Tukeys Trimean*

**Table 4:** Np-promoters’s for MC

| **ensembl_gene_id** | **external_gene_name** | **TM_Diff_CCC** | **TM_Diff_HGSC** | **TM_Diff_EC** | **nCpG** | **sigCpG** |
| --- | --- | --- | --- | --- | --- | --- |
| ENSG00000183486 | MX2 | 0.248391885708343 | 0.203352828260265 | 0.210776618829029 | 11 | 5 |
| ENSG00000101680 | LAMA1 | 0.227200522554289 | 0.195977383331007 | 0.230645845094763 | 6 | 3 |
| ENSG00000198838 | RYR3 | 0.147852418412696 | 0.148787308323006 | 0.136689311190024 | 11 | 3 |
| ENSG00000144229 | THSD7B | 0.140686134616983 | 0.164064303056023 | 0.146769792332137 | 7 | 3 |
| ENSG00000180644 | PRF1 | 0.216432522517782 | 0.212450581582084 | 0.192740667988718 | 10 | 5 |
| ENSG00000173431 | RNASE8 | 0.240610096883089 | 0.24592357832158 | 0.25276578265123 | 5 | 3 |
| ENSG00000198643 | FAM3D | 0.215313659894767 | 0.226048356675456 | 0.218422233679161 | 5 | 2 |
| ENSG00000156127 | BATF | 0.303616326378851 | 0.2598919896329 | 0.297193964242017 | 12 | 6 |
| ENSG00000124588 | NQO2 | 0.241198092145656 | 0.223366305696421 | 0.215834354594955 | 4 | 3 |
| ENSG00000182256 | GABRG3 | 0.196332583001122 | 0.168022530578287 | 0.203569910535991 | 15 | 5 |
| ENSG00000148848 | ADAM12 | 0.228070989315665 | 0.180027597311457 | 0.272983091865906 | 13 | 5 |
| ENSG00000134398 | ERN2 | 0.217488705526102 | 0.192914360421454 | 0.169922335769209 | 12 | 3 |
| ENSG00000196263 | ZNF471 | 0.195016543267368 | 0.195813460600208 | 0.195453975144572 | 9 | 4 |
| ENSG00000271698 |  | 0.203963625647674 | 0.188400468281872 | 0.185527323026242 | 11 | 3 |
| ENSG00000233041 | PHGR1 | 0.258073365357108 | 0.226900220054741 | 0.292527504479203 | 6 | 2 |
| ENSG00000198046 | ZNF667 | 0.171325035498943 | 0.149464526898559 | 0.216987494350133 | 6 | 2 |
| ENSG00000197273 | GUCA2A | 0.20933887974671 | 0.18026664482052 | 0.186084249463049 | 10 | 4 |
| ENSG00000134193 | REG4 | 0.219334481369786 | 0.214951050969996 | 0.208362834934423 | 7 | 4 |
| ENSG00000146674 | IGFBP3 | 0.0864674529858995 | 0.0881193106991515 | 0.101845614515362 | 22 | 6 |
| ENSG00000160182 | TFF1 | 0.254850391372846 | 0.226633977701592 | 0.22753722341765 | 10 | 5 |
| ENSG00000120057 | SFRP5 | 0.254013960822496 | 0.221622421937417 | 0.256618639040496 | 9 | 5 |
| ENSG00000216588 | IGSF23 | 0.279204935445197 | 0.26079336649722 | 0.280766929234564 | 9 | 5 |
| ENSG00000177669 | MBOAT4 | 0.224159042419682 | 0.21386486204215 | 0.21744706840831 | 6 | 3 |
| ENSG00000121797 | CCRL2 | 0.269116665424026 | 0.287183596050231 | 0.304421783717927 | 11 | 3 |
| ENSG00000152467 | ZSCAN1 | 0.312840468694615 | 0.244002694197208 | 0.355120808581618 | 9 | 6 |
| ENSG00000113303 | BTNL8 | 0.255111053580653 | 0.257845866123573 | 0.286924843285295 | 8 | 4 |
| ENSG00000138075 | ABCG5 | 0.143427766066386 | 0.13298879479209 | 0.162086386946243 | 10 | 3 |
| ENSG00000187258 | NPSR1 | 0.245797901975543 | 0.24472908804773 | 0.2596598692866 | 7 | 4 |
| ENSG00000132164 | SLC6A11 | 0.160338363411587 | 0.148152524339823 | 0.169632237727522 | 14 | 4 |
| ENSG00000171747 | LGALS4 | 0.351353062298097 | 0.286001805535721 | 0.306664898700844 | 7 | 5 |
| ENSG00000167117 | ANKRD40CL | 0.153450475417771 | 0.148551248236534 | 0.134855450989862 | 6 | 3 |
| ENSG00000163295 | ALPI | 0.23938839921772 | 0.280888929819079 | 0.261132696966984 | 5 | 3 |
| ENSG00000144820 | ADGRG7 | 0.141923174206722 | 0.16352824234822 | 0.171183266654298 | 4 | 2 |
| ENSG00000112419 | PHACTR2 | 0.0826460270936927 | 0.0769456597934922 | 0.0923552652370247 | 8 | 2 |
| ENSG00000178934 | LGALS7B | 0.190172555063573 | 0.181191245959134 | 0.206475772796828 | 5 | 2 |
| ENSG00000266200 | PNLIPRP2 | 0.417118588919226 | 0.398158641926161 | 0.498921802860366 | 3 | 3 |
| ENSG00000162572 | SCNN1D | 0.0995117341869036 | 0.0898667723493052 | 0.0986476473836149 | 8 | 2 |
| ENSG00000152672 | CLEC4F | 0.185189486574971 | 0.201196796392504 | 0.237280147514231 | 6 | 3 |
| ENSG00000162391 | FAM151A | 0.176682299277795 | 0.118913386846409 | 0.150302977012636 | 8 | 2 |
| ENSG00000168243 | GNG4 | 0.299160094723896 | 0.236415651384592 | 0.130333358407432 | 6 | 2 |
| ENSG00000188257 | PLA2G2A | 0.275111740742211 | 0.356969312221804 | 0.402330751626778 | 9 | 3 |
| ENSG00000168899 | VAMP5 | 0.258929333282314 | 0.307780785778697 | 0.302856136384922 | 9 | 4 |
| ENSG00000142949 | PTPRF | 0.208612548958125 | 0.197052393153909 | 0.214313008679115 | 3 | 2 |
| ENSG00000160972 | PPP1R16A | 0.194453149534456 | 0.151526690766497 | 0.0916400093580855 | 8 | 2 |
| ENSG00000164400 | CSF2 | 0.250110083995615 | 0.1185212781981 | 0.197320024434613 | 9 | 3 |
| ENSG00000087116 | ADAMTS2 | 0.175061352913141 | 0.189683426490473 | 0.195075157336389 | 8 | 2 |
| ENSG00000159387 | IRX6 | 0.121263122802771 | 0.0912403403040556 | 0.115299154080012 | 8 | 2 |
| ENSG00000146776 | ATXN7L1 | 0.256697263313576 | 0.19871935599579 | 0.231443496242056 | 5 | 2 |
| ENSG00000263761 | GDF2 | 0.273099056070423 | 0.311577162780111 | 0.362052490690248 | 11 | 3 |
| ENSG00000159398 | CES5A | 0.170792641214721 | 0.177004769387232 | 0.267507941641955 | 8 | 4 |
| ENSG00000205667 | ARSH | 0.201971282719125 | 0.175073573673731 | 0.21519403935726 | 5 | 2 |
| ENSG00000187824 | TMEM220 | 0.379670125229901 | 0.234009721966099 | 0.179649570525445 | 5 | 2 |
| ENSG00000137726 | FXYD6 | 0.0880883030323613 | 0.12401981717687 | 0.173492686973935 | 7 | 2 |
| ENSG00000091490 | SEL1L3 | 0.178819985143169 | 0.0469313534319555 | 0.063160422556451 | 8 | 2 |

*NP-PROMOTERs for MC. All NP-PROMOTERs have n > 33% CpGs with Δ β > 0.2 and adjusted p < 0.05 and aggregated p-value of p<0.05 for all three histotype comparisons. Tm_Diff: The TM of the NP-PROMOTER region, based on the TM of differences between the NP-PROMOTER group and the column histotype for each individual CpG site. NP-PROMOTER: Histotype specific promoter, nCpG: Number of CpG sites found overlapping with the genomic coordinates of the NP-PROMOTER region, sigCpG: Number of CpG sites deened statistically and biologically significant within the NP-PROMOTER region, CCC: Clear Cell Carcinoma, EC: Endometroid Carcinoma, HGSC: High Grade Serous Carcinoma, MC: Mucinous Carcinoma, TM: Tukeys Trimean*

**Table 5:** Percent of CpG’s in CCC NP-PROMOTER region associated with methylation state

| **Gene** | | | **CCC (% hemi)** | | | **EC (% hypo)** | | **HGSC (% hypo)** | | | **MC (% hypo)** | |
| --- | --- | --- | --- | --- | --- | --- | --- | --- | --- | --- | --- | --- |
| ENSG00000006025 | | | 0.333333333333333 | | | 0.666666666666667 | | 0.333333333333333 | | | 0.666666666666667 | |
| ENSG00000135392 | | | 0.5 | | | 0.666666666666667 | | 0.833333333333333 | | | 1 | |
| ENSG00000139914 | | | 0.75 | | | 0 | | 0 | | | 0 | |
| ENSG00000184956 | | | 0.272727272727273 | | | 0.181818181818182 | | 0.0909090909090909 | | | 0.0909090909090909 | |
| ENSG00000106384 | | | 0.666666666666667 | | | 0.583333333333333 | | 0.583333333333333 | | | 0.583333333333333 | |
| ENSG00000132128 | | | 0.444444444444444 | | | 0.777777777777778 | | 0.777777777777778 | | | 0.888888888888889 | |
| ENSG00000168439 | | 0.375 | | | 0.75 | | | | 0.625 | | | 0.75 |
| ENSG00000213965 | | 0.125 | | | 0.875 | | | | 0.75 | | | 0.75 |
| ENSG00000175414 | | | 0.25 | | | 1 | | 1 | | | 1 | |
| ENSG00000245848 | 0.666666666666667 | | | 1 | | | 1 | | | 1 | | |
| ENSG00000073712 | 0.333333333333333 | | | 0.5 | | | 0.5 | | | 0.666666666666667 | | |
| ENSG00000161653 | 0.75 | | | 0.666666666666667 | | | 0.833333333333333 | | | 0.75 | | |
| ENSG00000237172 | 0.714285714285714 | | | 0.714285714285714 | | | 0.714285714285714 | | | 0.714285714285714 | | |
| ENSG00000187554 | 0.6 | | | 0.9 | | | 1 | | | 1 | | |
| ENSG00000183560 | 1 | | | 0.2 | | | 0 | | | 0.2 | | |
| ENSG00000167701 | 1 | | | 0.5 | | | 1 | | | 0.5 | | |
| ENSG00000124818 | 0.375 | | | 0.375 | | | 0.375 | | | 0.375 | | |
| ENSG00000249481 | 0.888888888888889 | | | 0.666666666666667 | | | 0.555555555555556 | | | 0.888888888888889 | | |
| ENSG00000106336 | 0.5 | | | 0.125 | | | 0.125 | | | 0.125 | | |
| ENSG00000187800 | 0.6 | | | 0.8 | | | 1 | | | 0.8 | | |
| ENSG00000181409 | | | 0.6 | | | 0.6 | | 0.6 | | | 0.6 | |
| ENSG00000174600 | | | 0.9 | | | 0.7 | | 0.8 | | | 0.8 | |
| ENSG00000100253 | | | 0.636363636363636 | | | 0 | | 0 | | | 0.0909090909090909 | |
| ENSG00000183773 | | | 0.571428571428571 | | | 0.571428571428571 | | 0.571428571428571 | | | 0.571428571428571 | |
| ENSG00000269404 | 0.428571428571429 | | | 0.428571428571429 | | | 0.428571428571429 | | | 0.428571428571429 | | |
| ENSG00000285218 | | | 0.75 | | | 1 | | 1 | | | 1 | |
| ENSG00000013297 | | | 0.75 | | | 1 | | 1 | | | 1 | |
| ENSG00000174326 | | | 0.857142857142857 | | | 0.857142857142857 | | 0.857142857142857 | | | 0.857142857142857 | |
| ENSG00000131096 | | | 0.9375 | | | 0.4375 | | 1 | | | 0.9375 | |
| ENSG00000073067 | | | 0.555555555555556 | | | 0 | | 0.111111111111111 | | | 0.111111111111111 | |
| ENSG00000171124 | | | 0.375 | | | 0 | | 0 | | | 0 | |
| ENSG00000128567 | | | 0.142857142857143 | | | 0.714285714285714 | | 0.714285714285714 | | | 0.714285714285714 | |
| ENSG00000110696 | 0.571428571428571 | | | 0.857142857142857 | | | 0.857142857142857 | | | 0.857142857142857 | | |
| ENSG00000139154 | | | 0.6 | | | 0 | | 0 | | | 0 | |
| ENSG00000075651 | 0.714285714285714 | | | 0.142857142857143 | | | 0.142857142857143 | | | 0.142857142857143 | | |

*Percent of CpGs within NP-PROMOTER region in a specific methylation state. Columns shown represent the most frequent methylation type for histotype. Hypo: β < 0.3, Hemi: 0.3 < β < 0.7, Hyper: β >0.7, CCC: Clear Cell Carcinoma, EC: Endometroid Carcinoma, HGSC: High Grade Serous Carcinoma, MC: Mucinous Carcinoma, TM: Tukeys Trimean*

**Table 6:** Percent of CpG’s in HGSC NP-PROMOTER region associated with methylation state

| **Gene** | | | | | | | | | | | | | | | | | | | | **HGSC (% hyper)** | | | | | | **HGSC (% hypo)** | **CCC (% hyper)** | | **CCC (% hemi)** | | | | **EC (% hyper)** | | | **EC (% hemi)** | **MC (% hyper)** | | | | | **MC (% hemi)** | | | | | | | | | |
| --- | --- | --- | --- | --- | --- | --- | --- | --- | --- | --- | --- | --- | --- | --- | --- | --- | --- | --- | --- | --- | --- | --- | --- | --- | --- | --- | --- | --- | --- | --- | --- | --- | --- | --- | --- | --- | --- | --- | --- | --- | --- | --- | --- | --- | --- | --- | --- | --- | --- | --- | --- |
| ENSG00000108018 | | | | | | | | | | | | | | | | | | | | 0.25 | | | | | | 0.583333333333333 | 0.25 | | 0.583333333333333 | | | | 0.25 | | | 0.166666666666667 | 0.25 | | | | | 0.583333333333333 | | | | | | | | | |
| ENSG00000184937 | | | | | | | | | | | | | | | | | | | | 0 | | | | | | 0.666666666666667 | 0 | | 0.333333333333333 | | | | 0 | | | 0.333333333333333 | 0 | | | | | 0.333333333333333 | | | | | | | | | |
| ENSG00000145864 | | | | | | | | | | | | | | | | | | | | 0 | | | | | | 1 | 0 | | 0.375 | | | | 0 | | | 0 | 0 | | | | | 0.25 | | | | | | | | | |
| ENSG00000114638 | | | | | | | | | | | | | | | | | | | | 0.142857142857143 | | | | | | 0 | 0.285714285714286 | | 0.714285714285714 | | | | 0.428571428571429 | | | 0.571428571428571 | 0.285714285714286 | | | | | 0.714285714285714 | | | | | | | | | |
| ENSG00000110651 | 0.916666666666667 | | | | | 0.0833333333333333 | | | | | | 0.166666666666667 | | | | 0.833333333333333 | | | | | | | | 0.0833333333333333 | | | | | | | | 0.833333333333333 | | | | | | | | | | | 0.0833333333333333 | | | | | 0.916666666666667 | | | |
| ENSG00000140932 | 0.153846153846154 | | | | | 0 | | | | | | 0.153846153846154 | | | | 0.846153846153846 | | | | | | | | 0.153846153846154 | | | | | | | | 0.846153846153846 | | | | | | | | | | | 0.153846153846154 | | | | | 0.846153846153846 | | | |
| ENSG00000198626 | | | | | | | | | | | | | | | | | | | | 0 | | | | | | 0.333333333333333 | 0.166666666666667 | | 0.333333333333333 | | | | 0.166666666666667 | | | 0.5 | 0.333333333333333 | | | | | 0.666666666666667 | | | | | | | | | |
| ENSG00000158050 | | | 0 | 0.454545454545455 | | | | | 0.181818181818182 | | | | | 0.454545454545455 | | | | | | 0.0909090909090909 | | | | | | | | | | 0.454545454545455 | | | | | | | | | 0.272727272727273 | | | | | | | 0.363636363636364 | | | | | |
| ENSG00000092295 | | | | | | | | | | | | | | | | | | | | 0 | | | | | | 0 | 0.8 | | 0.2 | | | | 0.2 | | | 0.8 | 0.6 | | | | | 0.4 | | | | | | | | | |
| ENSG00000144837 | | | | | | | | | | | | | | | | | | | | 0.857142857142857 | | | | | | 0.142857142857143 | 0.142857142857143 | | 0.714285714285714 | | | | 0.428571428571429 | | | 0.428571428571429 | 0.571428571428571 | | | | | 0.285714285714286 | | | | | | | | | |
| ENSG00000124721 | | | | | | | | | | | | | | | | | | | | 0.25 | | | | | | 0.625 | 0.25 | | 0.25 | | | | 0.25 | | | 0.125 | 0.25 | | | | | 0.25 | | | | | | | | | |
| ENSG00000144407 | | | | | | | | | | | | | | | | | | | | 0.2 | | | | | | 0 | 1 | | 0 | | | | 0.8 | | | 0.2 | 1 | | | | | 0 | | | | | | | | | |
| ENSG00000180878 | | | | | | | | | | | | | | | | | | | | 0.5 | | | | | | 0 | 1 | | 0 | | | | 1 | | | 0 | 1 | | | | | 0 | | | | | | | | | |
| ENSG00000275410 | | | | | | | | | | | | | | | | | | | | 0 | | | | | | 0.909090909090909 | 0 | | 0 | | | | 0 | | | 0.0909090909090909 | 0 | | | | | 0 | | | | | | | | | |
| ENSG00000204396 | | | | | | | | | | | | | | | | | | | | 0.428571428571429 | | | | | | 0.19047619047619 | 0.80952380952381 | | 0 | | | | 0.80952380952381 | | | 0 | 0.80952380952381 | | | | | 0 | | | | | | | | | |
| ENSG00000184588 | | 0.222222222222222 | | | | | 0.555555555555556 | | | | | 0.222222222222222 | | | | | 0.333333333333333 | | | | | | | | 0.222222222222222 | | | | | | | 0.222222222222222 | | | | | | | | | | | | 0.222222222222222 | | | 0.666666666666667 | | | | |
| ENSG00000172995 | | 0.166666666666667 | | | | | 0.166666666666667 | | | | | 0.666666666666667 | | | | | 0.166666666666667 | | | | | | | | 0.666666666666667 | | | | | | | 0.166666666666667 | | | | | | | | | | | | 0.666666666666667 | | | 0.166666666666667 | | | | |
| ENSG00000196420 | | | | | | | | | | | | | | | | | | | | 0.25 | | | | | | 0 | 0.75 | | 0.25 | | | | 0.75 | | | 0.25 | 0.5 | | | | | 0.5 | | | | | | | | | |
| ENSG00000259900 | | | | | | | | | | | | | | | | | | | | 1 | | | | | | 0 | 0.875 | | 0.125 | | | | 0.875 | | | 0.125 | 0.25 | | | | | 0.75 | | | | | | | | | |
| ENSG00000157315 | | | | | | | | | | | | | | | | | | | | 1 | | | | | | 0 | 0.875 | | 0.125 | | | | 0.875 | | | 0.125 | 0.25 | | | | | 0.75 | | | | | | | | | |
| ENSG00000173212 | | | | | | | | | | | | | | | | | | | | 1 | | | | | | 0 | 0 | | 1 | | | | 0 | | | 1 | 0 | | | | | 1 | | | | | | | | | |
| ENSG00000124782 | | | | | 0.222222222222222 | | | | | | 0.777777777777778 | | | | | | | | 0 | | | | 0.222222222222222 | | | | | | | | | | | | 0 | | | 0.222222222222222 | | | | | | | | | | | 0 | | 0 |
| ENSG00000127903 | | | | | | | | | | | | | | | | | | | | 0 | | | | | | 0.375 | 0 | | 0.75 | | | | 0 | | | 0.625 | 0 | | | | | 1 | | | | | | | | | |
| ENSG00000103569 | | | 0.571428571428571 | | | | | 0 | | 1 | | | | | 0 | | | | | | 1 | | | | | | | | | | 0 | | | | | | | | | 1 | | | | | | 0 | | | | | |
| ENSG00000125533 | | | 0 | | | | | 0.7 | | 0 | | | | | 0.3 | | | | | | 0 | | | | | | | | | | 0.3 | | | | | | | | | 0 | | | | | | 0.6 | | | | | |
| ENSG00000188629 | | | 0.3 | | | | | 0.3 | | 0.4 | | | | | 0.4 | | | | | | 0.4 | | | | | | | | | | 0.3 | | | | | | | | | 0.4 | | | | | | 0.3 | | | | | |
| ENSG00000110079 | | | 0 | | | | | 0 | | 0.571428571428571 | | | | | 0.428571428571429 | | | | | | 0.571428571428571 | | | | | | | | | | 0.428571428571429 | | | | | | | | | 0.857142857142857 | | | | | | 0.142857142857143 | | | | | |
| ENSG00000206530 | | | 0.25 | | | | | 0 | | 0.416666666666667 | | | | | 0.583333333333333 | | | | | | 0.416666666666667 | | | | | | | | | | 0.583333333333333 | | | | | | | | | 0.333333333333333 | | | | | | 0.666666666666667 | | | | | |
| ENSG00000151365 | | | | | | | | | | | | | | | | | | | | 0.6 | | | | | | 0 | 1 | | 0 | | | | 0.8 | | | 0.2 | 1 | | | | | 0 | | | | | | | | | |
| ENSG00000197915 | | | | | | | | | | | | | 0.5 | | | | | 0 | | | | 1 | | | | | | 0 | | | | | | 1 | | | | | | | 0 | | | | 1 | | | | | 0 | |
| ENSG00000129467 | | | | | | | | | | | | | | | | | | | | 0.333333333333333 | | | | | | 0.333333333333333 | 0.333333333333333 | | 0.666666666666667 | | | | 0.333333333333333 | | | 0.333333333333333 | 0.333333333333333 | | | | | 0.666666666666667 | | | | | | | | | |

*Percent of CpGs within NP-PROMOTER region in a specific methylation state. Columns shown represent the most frequent methylation type for histotype. Hypo: β < 0.3, Hemi: 0.3 < β < 0.7, Hyper: β >0.7, CCC: Clear Cell Carcinoma, EC: Endometroid Carcinoma, HGSC: High Grade Serous Carcinoma, MC: Mucinous Carcinoma, TM: Tukeys Trimean*

**Table 7:** Percent of CpG’s in MC NP-PROMOTER region associated with methylation state

| **Gene** | **MC (% hemi/100)** | **CCC (% hyper/100)** | **EC (% hyper/100)** | **HGSC (% hyper/100)** |
| --- | --- | --- | --- | --- |
| ENSG00000183486 | 0.363636363636364 | 0.181818181818182 | 0.272727272727273 | 0.272727272727273 |
| ENSG00000101680 | 0.666666666666667 | 0.166666666666667 | 0.166666666666667 | 0.166666666666667 |
| ENSG00000198838 | 0.363636363636364 | 0 | 0 | 0 |
| ENSG00000144229 | 0.142857142857143 | 0 | 0 | 0 |
| ENSG00000180644 | 0.7 | 0.8 | 0.8 | 0.7 |
| ENSG00000173431 | 0.6 | 1 | 1 | 1 |
| ENSG00000198643 | 0.8 | 1 | 1 | 1 |
| ENSG00000156127 | 0.166666666666667 | 0.5 | 0.25 | 0.5 |
| ENSG00000124588 | 0.75 | 0.75 | 0.75 | 0.75 |
| ENSG00000182256 | 0.533333333333333 | 0.2 | 0.2 | 0.2 |
| ENSG00000148848 | 0.769230769230769 | 0.153846153846154 | 0.153846153846154 | 0.153846153846154 |
| ENSG00000134398 | 0.416666666666667 | 0.416666666666667 | 0.5 | 0.583333333333333 |
| ENSG00000196263 | 0.333333333333333 | 0 | 0 | 0 |
| ENSG00000271698 | 0.545454545454545 | 0.818181818181818 | 0.818181818181818 | 0.818181818181818 |
| ENSG00000233041 | 0.333333333333333 | 0.666666666666667 | 0.666666666666667 | 0.833333333333333 |
| ENSG00000198046 | 0.5 | 0 | 0 | 0 |
| ENSG00000197273 | 0.8 | 0.6 | 0.6 | 0.6 |
| ENSG00000134193 | 0.285714285714286 | 1 | 1 | 1 |
| ENSG00000146674 | 0.272727272727273 | 0 | 0 | 0 |
| ENSG00000160182 | 0.8 | 0.9 | 1 | 0.8 |
| ENSG00000120057 | 0.333333333333333 | 0.333333333333333 | 0.333333333333333 | 0.444444444444444 |
| ENSG00000216588 | 0.666666666666667 | 1 | 1 | 1 |
| ENSG00000177669 | 0.166666666666667 | 0.333333333333333 | 0.333333333333333 | 0.333333333333333 |
| ENSG00000121797 | 0.0909090909090909 | 0.181818181818182 | 0.181818181818182 | 0.181818181818182 |
| ENSG00000152467 | 0.777777777777778 | 0 | 0 | 0 |
| ENSG00000113303 | 0.625 | 1 | 1 | 1 |
| ENSG00000138075 | 0.4 | 0.9 | 0.9 | 0.9 |
| ENSG00000187258 | 0.714285714285714 | 0.857142857142857 | 0.857142857142857 | 0.857142857142857 |
| ENSG00000132164 | 0.285714285714286 | 0.214285714285714 | 0.214285714285714 | 0.214285714285714 |
| ENSG00000171747 | 0.857142857142857 | 0.857142857142857 | 0.714285714285714 | 0.857142857142857 |
| ENSG00000167117 | 0.833333333333333 | 0.5 | 0.5 | 0.5 |
| ENSG00000163295 | 0.8 | 1 | 1 | 1 |
| ENSG00000144820 | 0.25 | 1 | 1 | 0.75 |
| ENSG00000112419 | 0.5 | 0.25 | 0.25 | 0.25 |
| ENSG00000178934 | 0.8 | 0.8 | 0.8 | 0.8 |
| ENSG00000266200 | 1 | 1 | 0.666666666666667 | 1 |
| ENSG00000162572 | 0.125 | 1 | 1 | 1 |
| ENSG00000152672 | 0.666666666666667 | 0.833333333333333 | 0.833333333333333 | 1 |
| ENSG00000162391 | 0.125 | 0.75 | 0.5 | 0.75 |
| ENSG00000168243 | 0.166666666666667 | 0.333333333333333 | 0.333333333333333 | 0.166666666666667 |
| ENSG00000188257 | 0.555555555555556 | 0.444444444444444 | 0.777777777777778 | 0.777777777777778 |
| ENSG00000168899 | 0.111111111111111 | 0.222222222222222 | 0.222222222222222 | 0.333333333333333 |
| ENSG00000142949 | 1 | 0.666666666666667 | 0.333333333333333 | 0.333333333333333 |
| ENSG00000160972 | 0.375 | 0.5 | 0.5 | 0.375 |
| ENSG00000164400 | 0.666666666666667 | 0.888888888888889 | 0.666666666666667 | 0.888888888888889 |
| ENSG00000087116 | 0.625 | 0.125 | 0.125 | 0.25 |
| ENSG00000159387 | 0.5 | 0.125 | 0.125 | 0.125 |
| ENSG00000146776 | 0.6 | 0.6 | 0.6 | 0.6 |
| ENSG00000263761 | 0.818181818181818 | 0.727272727272727 | 0.818181818181818 | 0.909090909090909 |
| ENSG00000159398 | 0.625 | 0.625 | 0.625 | 0.75 |
| ENSG00000205667 | 0.6 | 1 | 0.8 | 0.6 |
| ENSG00000187824 | 0.6 | 0.8 | 0.6 | 0.4 |
| ENSG00000137726 | 0.714285714285714 | 0.428571428571429 | 0.571428571428571 | 0.571428571428571 |
| ENSG00000091490 | 0.75 | 0.5 | 0.375 | 0.375 |

*Percent of CpGs within NP-PROMOTER region in a specific methylation state. Columns shown represent the most frequent methylation type for histotype. Hypo: β < 0.3, Hemi: 0.3 < β < 0.7, Hyper: β >0.7, CCC: Clear Cell Carcinoma, EC: Endometroid Carcinoma, HGSC: High Grade Serous Carcinoma, MC: Mucinous Carcinoma, TM: Tukeys Trimean*

**Figure 7:** DMP results from Minfi and ChAMP

*DMP: Differentially methylated probe/CpG, Clear Cell Carcinoma, EC: Endometroid Carcinoma, HGSC: High Grade Serous Carcinoma, MC: Mucinous Carcinoma*

**Figure 8:** Percent overlap between DMP results

*DMP: Differentially methylated probe/CpG, Clear Cell Carcinoma, EC: Endometroid Carcinoma, HGSC: High Grade Serous Carcinoma, MC: Mucinous Carcinoma*

**Figure 9:** Number of DMRs found in different DMR callers

*DMR: Differentially methylated region, Clear Cell Carcinoma, EC: Endometroid Carcinoma, HGSC: High Grade Serous Carcinoma, MC: Mucinous Carcinoma*

**Figure 10:** Overlap between DMR caller results

*Unique overlaps of DMR regions found in one DMR-caller, compared to DMR regions for the same contrast in other DMR-callers. Percentage representative of the ratio of unique overlaps in the other DMR caller, to that of the total number of DMRs in the reference DMR caller. CCC: Clear Cell Carcinoma, EC: Endometroid Carcinoma, HGSC: High Grade Serous Carcinoma, MC: Mucinous Carcinoma*

**Figure 11:** Distribution type of significant np-promoter CpGs not found in DMP caller results for ChAMP

*Normal: Shapiro-Wilks p > 0.05, NotNormal: Shapiro-Wilks p < 0.05. CCC: Clear Cell Carcinoma, EC: Endometroid Carcinoma, HGSC: High Grade Serous Carcinoma, MC: Mucinous Carcinoma*

**Figure 12:** Distribution type of significant np-promoter CpGs not found in DMP caller results for minfi

*Normal: Shapiro-Wilks p > 0.05, NotNormal: Shapiro-Wilks p < 0.05. CCC: Clear Cell Carcinoma, EC: Endometroid Carcinoma, HGSC: High Grade Serous Carcinoma, MC: Mucinous Carcinoma*

**Figure 13:** Differences between Brunner-Munz and t-test for np-promoter CpGs using β Values, CCC as reference

*Differences in* Δβ when using trimean and mean for CpG-sites in NP-PROMOTERs. *Normal: Shapiro-Wilks p > 0.05, NotNormal: Shapiro-Wilks p < 0.05. CCC: Clear Cell Carcinoma, EC: Endometroid Carcinoma, HGSC: High Grade Serous Carcinoma, MC: Mucinous Carcinoma*

**Figure 14:** Differences between Tukeys test and Dunns-test for np-promoter CpGs using β Values, CCC as reference

**Figure 15:** Flowchart for the sequential model selection,

*Flowchart for the sequential model selection, white boxes at left corner illustrate the model after n=1-3 iterations, where CGX_g1_ … CGC_g3_ represent the CpG C for gene G1 … G3, NP-Promoter: Uniquely differentially methylated promoter region characterized by the non-parametric methodology in manuscript. Histotype specific promoter, CV: Cross validation, CCC: Clear Cell Carcinoma, EC: Endometroid Carcinoma, HGSC: High Grade Serous Carcinoma, MC: Mucinous Carcinoma*

**Table 12:** Predictive classification statistics for multiclass model

| **GEO ID** | **CCC Sensitivity** | **HGSC Sensitivity** | **MC Sensitivity** | **CCC Specificity** | **HGSC Specificity** | **MC Specificity** |
| --- | --- | --- | --- | --- | --- | --- |
| *GSE51820* | 0.769 | 0.867781818181818 | 0.613 | 0.958252106227106 | 0.810259523809524 | 0.974062114845938 |
| *GSE226823* | 0.892035714285714 | 0.934777777777778 | 0 | 0.981790909090909 | 0.95575 | 0.97795572755418 |

*CCC: Clear Cell Carcinoma, EC: Endometroid Carcinoma, HGSC: High Grade Serous Carcinoma, MC: Mucinous Carcinoma*

**Table 13:** Predictive classification statistics for multiclass model

| **GEO ID** | **CCC F1** | **HGSC F1** | **MC F1** | **Overall Accuracy** | **AUC** | **PR AUC** |
| --- | --- | --- | --- | --- | --- | --- |
| *GSE51820* | 0.757104761904762 | 0.875017069709728 | 0.6001254555066231 | 0.799863923288614 | 0.825694023569024 | 0.703092920698361 |
| *GSE226823* | 0.927471693012869 | 0.944460550663647 | 0 | 0.890141864465084 | 0.876658134920635 | 0.79069162346457 |

*CCC: Clear Cell Carcinoma, EC: Endometroid Carcinoma, HGSC: High Grade Serous Carcinoma, MC: Mucinous Carcinoma, PR: Precision Recall, AUC: Area under curve*

**Figure 16:** Binary classification statistics using multiclass model predictors for each histotype

 Statistics for binary classification of EOC histotypes using XGBoost in external cohorts GSE51820 and GSE226823 (using multiclass model predictors). Values in classification statistics are representative of the mean of 5-fold cross-validation, repeated over 100 iterations. CCC: Clear Cell Carcinoma, EC: Endometroid Carcinoma, HGSC: High Grade Serous Carcinoma, MC: Mucinous Carcinoma, SID: Gene Expression Omnibus (GEO) id, AUC: Area Under Curve, CI_ Range: Lower and upper bounds for confidence interval (95%), NP-PROMOTER: Histotype Specific Promoter

**Table 14:** Predictive classification statistics for binary classification (one vs. rest) using NP-PROMOTER multiclass model

| **GEO ID** | **CCC Sensitivity** | **HGSC Sensitivity** | **MC Sensitivity** | **CCC Specificity** | **HGSC Specificity** | **MC Specificity** |
| --- | --- | --- | --- | --- | --- | --- |
| *GSE51820* | 0.7692 | 0.849029900332226 | 0.625666666666667 | 0.847209921355112 | 0.718781538461538 | 1 |
| *226823* | 0.837818390804598 | 0.934771771771772 | NA | 0.907394291754757 | 0.911111111111111 | NA |

*CCC: Clear Cell Carcinoma, EC: Endometroid Carcinoma, HGSC: High Grade Serous Carcinoma, MC: Mucinous Carcinoma*

**Table 15:** Predictive classification statistics for binary classification (one vs. rest) using NP-PROMOTER multiclass model

| **GEO ID** | **CCC F1** | **HGSC F1** | **MC F1** | **CCC AUC** | **HGSC AUC** | **MC AUC** | **CCC PR AUC** | **HGSC PR AUC** | **MC PR AUC** |
| --- | --- | --- | --- | --- | --- | --- | --- | --- | --- |
| *GSE51820* | 0.701831933525803 | 0.841027216246487 | 0.765739393939394 | 0.840303096298741 | 0.830240528154016 | 0.919706945178918 | 0.705554952433114 | 0.839503918641169 | 0.841227627617696 |
| *GSE226823* | 0.849236911910523 | 0.924722899565792 | NA | 0.93391961920731 | 0.951431556556557 | NA | 0.929445286122701 | 0.932355011308704 | NA |

*CCC: Clear Cell Carcinoma, EC: Endometroid Carcinoma, HGSC: High Grade Serous Carcinoma, MC: Mucinous Carcinoma, PR: Precision Recall, AUC: Area under curve*

**Table 16:** OncoScore results from CCC np-promoter regions

| **Gene** | **Percent Citation** | **Oncoscore** |
| --- | --- | --- |
| MUC6 | 79.9658994032396 | 72.1230199384229 |
| CYP2W1 | 75.3086419753086 | 63.430026244488 |
| LRRC41 | 88.8888888888889 | 60.8475665079352 |
| CEBPA | 64.4792548687553 | 58.725154874788 |
| FERMT2 | 55.3333333333333 | 47.6787862071088 |
| AIFM3 | 51.7241379310345 | 41.0768879761844 |
| ARL10 | 66.6666666666667 | 40.8764795176972 |
| AEBP2 | 49.1525423728814 | 40.7970222503042 |
| STIP1 | 45.5284552845528 | 40.1894253168149 |
| FUT3 | 45.0757575757576 | 39.4723824915751 |
| SPIB | 44.5355191256831 | 39.3057059604204 |
| OSBPL7 | 54.5454545454545 | 38.7782822008425 |
| FBXO24 | 50 | 38.0093766715934 |
| PODXL | 35.3409090909091 | 31.7278215319225 |
| CMKLR1 | 35.5056179775281 | 31.4698162052972 |
| AATK | 38 | 31.2670148348488 |
| PLD1 | 31.8525519848771 | 28.6822365529681 |
| CLDN11 | 32.258064516129 | 28.2874117799175 |
| SPATS1 | 36.3636363636364 | 25.852188133895 |
| TLR5 | 25.7774140752864 | 23.487112108003 |

**Table 17:** OncoScore results from CCC np-promoter regions

| **Gene** | **Percent Citation** | **Oncoscore** |
| --- | --- | --- |
| ZNF177 | 100 | 69.8970004336019 |
| WT1 | 71.4812538414259 | 65.9784318567921 |
| RREB1 | 71.4876033057851 | 62.4600950430728 |
| C11orf42 | 100 | 50 |
| TMED6 | 70 | 48.9279003035213 |
| VWA7 | 60 | 41.9382002601611 |
| S100A5 | 50 | 41.2287468209023 |
| UPK1B | 48 | 40.7752801040644 |
| ADCY4 | 47.4576271186441 | 39.3902283796041 |
| CMTM2 | 45.9459459459459 | 37.1262209726716 |
| CD81 | 39.8161244695898 | 36.3434548199781 |
| MS4A4A | 41.9047619047619 | 35.6635897887729 |
| DUSP2 | 40.3571428571429 | 35.3927268350877 |
| HNF1B | 38.9105058365759 | 35.2364407773695 |
| HRNR | 40 | 33.6728163624087 |
| DNAH8 | 36.734693877551 | 30.1921128898527 |
| PLA1A | 30.7692307692308 | 25.3715365051706 |
| PDE4B | 27.4336283185841 | 24.5841313811774 |
| BHLHE23 | 40 | 22.7729376770643 |
| ARPP21 | 22.2222222222222 | 18.504440470885 |

**Table 18:** OncoScore results from MC np-promoter regions

| **Gene** | **Percent Citation** | **Oncoscore** |
| --- | --- | --- |
| TFF1 | 87.2727272727273 | 78.8036937302074 |
| TMEM220 | 94.1176470588235 | 71.0917136829905 |
| ZNF471 | 93.75 | 70.3125 |
| REG4 | 77.3869346733668 | 67.2532776562097 |
| ZNF667 | 78.9473684210526 | 66.3115994798825 |
| GNG4 | 72.3076923076923 | 60.3011701966937 |
| PHGR1 | 75 | 54.0792790761653 |
| TNFRSF5 | 67.741935483871 | 54.0682876831481 |
| GUCA2A | 62 | 51.0146031515954 |
| ADGRG7 | 65.2173913043478 | 50.800126339728 |
| FAM3D | 60.8695652173913 | 49.8495810655628 |
| ZSCAN1 | 66.6666666666667 | 49.6027983460123 |
| BATF | 51.4583333333333 | 45.6809711011415 |
| PHACTR2 | 56.25 | 45 |
| LGALS4 | 50.8982035928144 | 44.8271159426327 |
| NQO2 | 50.6849315068493 | 44.496162609243 |
| SFRP5 | 48.9411764705882 | 43.335932143049 |
| PTPRF | 49.4047619047619 | 42.7215042631831 |
| ADAM12 | 46.9416785206259 | 41.9781818484659 |
| SEL1L3 | 51.8518518518519 | 40.9468931481477 |
| FXYD6 | 49.1803278688525 | 40.8878803923196 |
| THSD7B | 52 | 40.8024094900918 |
| BTNL8 | 48.3870967741936 | 38.6202054879629 |
| PLA2G2A | 43.3333333333333 | 38.2988648333722 |
| PRF1 | 42.6211453744493 | 38.2837989064018 |
| CSF2 | 42.002442002442 | 37.6623243344621 |
| LGALS7B | 75 | 37.5 |
| CCRL2 | 41.1111111111111 | 35.6236688745232 |
| IGFBP3 | 34.5218800648298 | 31.8818824632087 |
| LAMA1 | 36.4928909952607 | 31.7665055281973 |
| PNLIPRP2 | 37.5 | 28.125 |
| ERN2 | 31.1881188118812 | 27.5859754738475 |
| ADAMTS2 | 28.8372093023256 | 25.1154112182919 |
| ATXN7L1 | 37.5 | 25 |
| CLEC4F | 29.4117647058824 | 24.2267225179741 |
| GDF2 | 26.8170426065163 | 23.7133062188947 |
| VAMP5 | 28.2608695652174 | 23.1444483518684 |
| FAM151A | 40 | 22.7729376770643 |
| MX2 | 23.841059602649 | 21.5124683611759 |

**Table 19:** Standard Deviance from TM in training cohort for CCC NP-PROMOTERs present in EPIC, 450K arrays

| **Gene** | **Max SD NP-PROMOTER** | **Mean SD NP-PROMOTER** |
| --- | --- | --- |
| ENSG00000213965 | 0.0162578555070172 | 0.00819261915833197 |
| ENSG00000171124 | 0.034467087016043 | 0.0250397941790091 |
| ENSG00000237172 | 0.036766900323117 | 0.0233069861718783 |
| ENSG00000183560 | 0.042913422679579 | 0.0300519494089444 |
| ENSG00000073712 | 0.048168020680729 | 0.0236815801171945 |
| ENSG00000139914 | 0.051438976658057 | 0.0397801110064355 |
| ENSG00000135392 | 0.0523870799230385 | 0.0523870799230385 |
| ENSG00000174326 | 0.05380315116153 | 0.0275177004120328 |
| ENSG00000285218 | 0.065441617221165 | 0.0317336063774741 |
| ENSG00000013297 | 0.065441617221165 | 0.0317336063774741 |
| ENSG00000167701 | 0.066120000154268 | 0.0533104213484048 |
| ENSG00000168439 | 0.0667403004197125 | 0.0667403004197125 |
| ENSG00000006025 | 0.0758466933277245 | 0.0246249566587497 |
| ENSG00000073067 | 0.0822312667053875 | 0.0353458461502739 |
| ENSG00000187554 | 0.0874304452537645 | 0.0148907812706043 |
| ENSG00000075651 | 0.0883152671579055 | 0.0836756479880418 |
| ENSG00000106336 | 0.092455757692328 | 0.092455757692328 |
| ENSG00000183773 | 0.100509690772119 | 0.0697047257074181 |
| ENSG00000184956 | 0.103756460785187 | 0.0384566962859096 |
| ENSG00000110696 | 0.106163574067596 | 0.0392289585191946 |
| ENSG00000100253 | 0.108083309608859 | 0.0574761715706283 |
| ENSG00000124818 | 0.109299979960384 | 0.026583549885765 |
| ENSG00000128567 | 0.132105694179607 | 0.0534955556312925 |
| ENSG00000187800 | 0.140550346709343 | 0.0429546371906986 |
| ENSG00000131096 | 0.140754817850096 | 0.0921657404601121 |
| ENSG00000161653 | 0.140754817850096 | 0.0798166029527661 |
| ENSG00000249481 | 0.152481769775848 | 0.0763249873883676 |
| ENSG00000174600 | 0.155268760899736 | 0.0941401529317081 |
| ENSG00000269404 | 0.157865457047147 | 0.036934344922288 |
| ENSG00000181409 | 0.158690133465426 | 0.0642780031805404 |
| ENSG00000106384 | 0.167574022621314 | 0.0814427248300456 |
| ENSG00000245848 | 0.176632850575605 | 0.0571966977513678 |
| ENSG00000139154 | 0.190119053217068 | 0.136362235263954 |
| ENSG00000175414 | 0.212714494003195 | 0.0829077629613239 |
| ENSG00000132128 | 0.228237786956628 | 0.185659958824077 |

*Standard deviance of NP-PROMOTERs against external cohorts, for NP-PROMOTERs with n>=3 CpG’s in both the EPIC and 450K array. Max SD NP-PROMOTER; Largest observed SD for a CpG site in the NP-PROMOTER, in all external cohorts, Mean SD NP-PROMOTER; Mean SD for CpG sites in the NP-PROMOTER, SD; Standard deviance*

**Table 20:** Standard Deviance from TM in training cohort for HGSC NP-PROMOTERs

| **Gene** | **Max SD NP-PROMOTER** | **Mean SD NP-PROMOTER** |
| --- | --- | --- |
| ENSG00000184937 | 0.0225813694220811 | 0.0086830951083823 |
| ENSG00000125533 | 0.0251649197702666 | 0.0174141472136522 |
| ENSG00000259900 | 0.0265048018632466 | 0.0203549532213925 |
| ENSG00000157315 | 0.0265048018632466 | 0.0203549532213925 |
| ENSG00000145864 | 0.0320864749943826 | 0.00959540801910812 |
| ENSG00000188629 | 0.0378731144861178 | 0.0249520465590702 |
| ENSG00000129467 | 0.0382872280992229 | 0.0207788619296338 |
| ENSG00000184588 | 0.0431439417909442 | 0.0200655172136509 |
| ENSG00000124721 | 0.0481257380783622 | 0.0106803266449387 |
| ENSG00000127903 | 0.0559328979592073 | 0.0288897170089665 |
| ENSG00000108018 | 0.0559665608743534 | 0.014830884118292 |
| ENSG00000114638 | 0.0645790608592905 | 0.0448792888932537 |
| ENSG00000198626 | 0.066063527681542 | 0.0209950256211298 |
| ENSG00000158050 | 0.0673439815248082 | 0.0190595244784591 |
| ENSG00000110651 | 0.0708070652910632 | 0.0255486677736989 |
| ENSG00000144407 | 0.0763532000536783 | 0.071737326163183 |
| ENSG00000172995 | 0.0796613660776978 | 0.0572946698509341 |
| ENSG00000204396 | 0.0810074237844165 | 0.0336479347601945 |
| ENSG00000092295 | 0.0828001118658812 | 0.0740507823448898 |
| ENSG00000144837 | 0.0925265926784324 | 0.0335802671282448 |
| ENSG00000196420 | 0.0929414743830732 | 0.0544319737053838 |
| ENSG00000206530 | 0.0936317998865268 | 0.0445270025044178 |
| ENSG00000180878 | 0.10307661146531 | 0.074994037410851 |
| ENSG00000275410 | 0.116525068061565 | 0.0880108062259712 |
| ENSG00000197915 | 0.121749556349491 | 0.078925960866504 |
| ENSG00000124782 | 0.129226311187181 | 0.02487490769832 |
| ENSG00000140932 | 0.14879564716985 | 0.0722193670900072 |
| ENSG00000110079 | 0.149119650044403 | 0.100405221738392 |
| ENSG00000151365 | 0.155843715868955 | 0.0530109635546258 |
| ENSG00000173212 | 0.162619487107198 | 0.0561208902866321 |
| ENSG00000103569 | 0.187574398365286 | 0.142538980219243 |

*Standard deviance of NP-PROMOTERs against external cohorts, for NP-PROMOTERs with n>=3 CpG’s in both the EPIC and 450K array. Max SD NP-PROMOTER; Largest observed SD for a CpG site in the NP-PROMOTER, in all external cohorts, Mean SD NP-PROMOTER; Mean SD for CpG sites in the NP-PROMOTER, SD; Standard deviance*

**Table 21:** Standard Deviance from TM in training cohort for MC NP-PROMOTERs present in EPIC, 450K arrays

| **Gene** | **Max SD NP-PROMOTER** | **Mean SD NP-PROMOTER** |
| --- | --- | --- |
| ENSG00000138075 | ENSG00000138075 | 0.0341773240993588 |
| ENSG00000146776 | ENSG00000146776 | 0.026737728181325 |
| ENSG00000178934 | ENSG00000178934 | 0.0157618076930692 |
| ENSG00000233041 | ENSG00000233041 | 0.0335801221128618 |
| ENSG00000171747 | ENSG00000171747 | 0.0554520732143022 |
| ENSG00000144820 | ENSG00000144820 | 0.084753220509126 |
| ENSG00000160182 | ENSG00000160182 | 0.0562614298994313 |
| ENSG00000152467 | ENSG00000152467 | 0.0347013839410142 |
| ENSG00000091490 | ENSG00000091490 | 0.0963446383117192 |
| ENSG00000113303 | ENSG00000113303 | 0.0884923900551281 |
| ENSG00000087116 | ENSG00000087116 | 0.0474221681508981 |
| ENSG00000121797 | ENSG00000121797 | 0.0366464146596048 |
| ENSG00000120057 | ENSG00000120057 | 0.0558889417467396 |
| ENSG00000112419 | ENSG00000112419 | 0.0270044515199013 |
| ENSG00000168899 | ENSG00000168899 | 0.0389790257409671 |
| ENSG00000134398 | ENSG00000134398 | 0.0581044935252324 |
| ENSG00000188257 | ENSG00000188257 | 0.0767292865445942 |
| ENSG00000180644 | ENSG00000180644 | 0.070464153150824 |
| ENSG00000266200 | ENSG00000266200 | 0.108808883608562 |
| ENSG00000173431 | ENSG00000173431 | 0.0811923495906884 |
| ENSG00000177669 | ENSG00000177669 | 0.0406909729986352 |
| ENSG00000168243 | ENSG00000168243 | 0.0847711688401546 |
| ENSG00000124588 | ENSG00000124588 | 0.111925145983613 |
| ENSG00000216588 | ENSG00000216588 | 0.146712574875661 |
| ENSG00000198643 | ENSG00000198643 | 0.0878231290028576 |
| ENSG00000271698 | ENSG00000271698 | 0.0765431117030397 |
| ENSG00000160972 | ENSG00000160972 | 0.0463559533614735 |
| ENSG00000183486 | ENSG00000183486 | 0.0435687227441388 |
| ENSG00000167117 | ENSG00000167117 | 0.136697134345787 |
| ENSG00000144229 | ENSG00000144229 | 0.0905024744648227 |
| ENSG00000159387 | ENSG00000159387 | 0.121372403282595 |
| ENSG00000132164 | ENSG00000132164 | 0.0718768164758062 |
| ENSG00000164400 | ENSG00000164400 | 0.104439168171239 |
| ENSG00000205667 | ENSG00000205667 | 0.134978214782592 |
| ENSG00000187258 | ENSG00000187258 | 0.125102738430703 |
| ENSG00000198838 | ENSG00000198838 | 0.0847173679603608 |
| ENSG00000197273 | ENSG00000197273 | 0.0746130661390021 |
| ENSG00000156127 | ENSG00000156127 | 0.148798948204486 |
| ENSG00000101680 | ENSG00000101680 | 0.0544315025093415 |
| ENSG00000187824 | ENSG00000187824 | 0.105298929739238 |
| ENSG00000263761 | ENSG00000263761 | 0.142758709146767 |
| ENSG00000163295 | ENSG00000163295 | 0.195420738558618 |
| ENSG00000196263 | ENSG00000196263 | 0.090249566389676 |
| ENSG00000198046 | ENSG00000198046 | 0.104472217012461 |
| ENSG00000152672 | ENSG00000152672 | 0.116317200366982 |
| ENSG00000134193 | ENSG00000134193 | 0.199240579804791 |
| ENSG00000137726 | ENSG00000137726 | 0.125526061423916 |
| ENSG00000162391 | ENSG00000162391 | 0.0438059776549321 |
| ENSG00000162572 | ENSG00000162572 | 0.303615857049602 |
| ENSG00000182256 | ENSG00000182256 | 0.104936722260956 |
| ENSG00000148848 | ENSG00000148848 | 0.0421653148352689 |
| ENSG00000146674 | ENSG00000146674 | 0.0756117480294167 |
